# Assessing the Functional Relevance of Variants in the *IKAROS Family Zinc Finger Protein 1* (*IKZF1*) in a Cohort of Patients With Primary Immunodeficiency

**DOI:** 10.3389/fimmu.2019.00568

**Published:** 2019-04-16

**Authors:** Zoya Eskandarian, Manfred Fliegauf, Alla Bulashevska, Michele Proietti, Rosie Hague, Cristian Roberto Smulski, Desirée Schubert, Klaus Warnatz, Bodo Grimbacher

**Affiliations:** ^1^Institute for Immunodeficiency, Center for Chronic Immunodeficiency, Medical Center, Faculty of Medicine, Albert-Ludwigs-University of Freiburg, Freiburg, Germany; ^2^Faculty of Biology, Albert-Ludwigs-University of Freiburg, Freiburg, Germany; ^3^Centre for Integrative Biological Signalling Studies, Albert-Ludwigs University of Freiburg, Freiburg, Germany; ^4^Royal Hospital for Children, Glasgow, United Kingdom; ^5^Department of Medical Physics, Centro Atómico Bariloche, CONICET, San Carlos de Bariloche, Argentina; ^6^Clinic for Rheumatology and Clinical Immunology, Faculty of Medicine, CCI, Medical Center, Albert-Ludwigs-University of Freiburg, Freiburg, Germany; ^7^Satellite Center Freiburg, RESIST–Cluster of Excellence 2155, Hanover Medical School, Freiburg, Germany; ^8^Satellite Center Freiburg, German Center for Infection Research, Freiburg, Germany; ^9^Institute of Immunity and Transplantation, Royal Free Hospital, University College London, London, United Kingdom

**Keywords:** CVID, monogenic defects, IKAROS, TRAIL, DNA binding, nuclear localization

## Abstract

Common variable immunodeficiency (CVID) is the most frequent symptomatic primary immunodeficiency. Patients with CVID are prone to recurrent bacterial infection due to the failure of adequate immunoglobulin production. Monogenetic defects have been identified in ~25% of CVID patients. Recently, mutations in *IKZF1*, encoding the zinc-finger transcription factor IKAROS which is broadly expressed in hematopoietic cells, have been associated with a CVID-like phenotype. Herein we describe 11 patients with heterozygous *IKZF1* variants from eight different families with autosomal dominant CVID and two siblings with an *IKZF1* variant presenting with inflammatory bowel disease (IBD). This study shows that mutations affecting the DNA binding domain of IKAROS can impair the interaction with the target DNA sequence thereby preventing heterochromatin and pericentromeric localization (HC-PC) of the protein. Our results also indicate an impairment of pericentromeric localization of IKAROS by overexpression of a truncated variant, caused by an immature stop codon in *IKZF1*. We also describe an additional variant in *TNFSF10*, encoding Tumor Necrosis Factor Related Apoptosis Inducing Ligand (TRAIL), additionally presented in individuals of Family A. Our results indicate that this variant may impair the TRAIL-induced apoptosis in target cell lines and prohibit the NFκB activation by TRAIL and may act as a modifier in Family A.

## Introduction

Common variable immunodeficiency (CVID) is the most frequent symptomatic primary immunodeficiency with an estimated incidence of 1:50,000–1:25,000. The disease is characterized by recurrent infections, due to a marked decrease in serum IgG commonly in association with reduced IgM and/or IgA. Most CVID-affected individuals have reduced numbers of isotype-switched memory B cells but relative preservation of pre-germinal center B cells (1–3). As a clinically and genetically heterogeneous disorder, CVID has a variable age of onset. In a recent study, an early disease onset (<10 years) was reported in 33.7% of the individuals in a cohort of 2,212 European CVID patients, however, the median diagnostic delay for all the patients in this study was 4.1 years [interquartile range [IQR], 1–11.8 years] (4). Although patients with CVID share many clinical and immunological features, the degree and severity of the phenotype varies considerably between affected individuals (5). In addition to symptoms of immune deficiency, 25% of CVID patients exhibit a significantly increased risk of autoimmunity (6, 7). In fact, some patients suffer of an immune dysregulation syndrome with autoimmunity, enteropathy, granulomatous disease, lymphoproliferation, and malignancy (8, 9).

To date, a monogenetic defect can be identified in up to 10–30% of CVID patients. There is a predominance of autosomal-dominant over recessive mode of inheritance (4, 10), but disease penetrance can be incomplete, or may appear to be so in some kindred due to the late onset of symptoms (4). Genomic approaches using Sanger sequencing, targeted gene panel sequencing, and more recently whole exome sequencing (WES), have identified mutations in several genes encoding proteins essential for immune function. These include *ICOS* (MIM: 607594), *CD19* (MIM: 613493), *CD81* (MIM: 613496), *MS4A1* (MIM: 613495), *CR2* (MIM: 614699), *TNFSF12* (MIM: 602695), *CTLA4* (MIM: 123890), *LRBA* (MIM: 614700) *TNFRSF13C* (MIM: 613494) *NFKB1*(MIM*:*164011), and *NFKB2* (OMIM: 615577) all of which may be causative for the CVID phenotype in affected individuals (11–15). Recently, mutations in *IKZF1*, encoding IKAROS, have been shown to be associated with a CVID-like phenotype (16). IKAROS is a zinc-finger transcription factor, broadly expressed in hematopoietic cells (17, 18). It binds to the regulatory regions of its target genes and interacts with chromatin remodeling complexes resulting in their conversion into pericentromeric heterochromatin (PC-HC) (19, 20). Apart from its role in T cell development (21), IKAROS has been shown to exert an important role during the early stages of B cell development, as homozygous mice from two distinct IKAROS-targeted mutations lack B cells (18, 22). Beyond this central role in early B cell development, IKAROS plays crucial roles in B lineage specification and commitment (18), as well as in immunoglobulin gene recombination. IKAROS is also an important regulator of B cell activation (23). Recently, it has been shown that the development and function of human dendritic cells (DCs) is regulated by IKAROS and heterozygous mutation of *IKZF1* in human can reduce the number of plasmacytoid dendritic cell (pDC) (24).

The human *IKZF1* is located at 7p12 and contains eight exons (25). Alternative splicing leads to the generation of at least eight IKAROS isoforms that confer complex functional diversity *in vivo* (22, 26). The basic structure of the longest IKAROS isoform (NM_006060.6; UniProt: gi|3913926) with 519 amino acids, consists of an N-terminal DNA binding domain with four centrally located C2H2 zinc fingers and a C-terminal domain with two additional C2H2 zinc fingers, which are important in dimerization and multimerization of the protein (27–29). The C2H2 zinc finger domain in IKAROS consists of three tandem zinc fingers which bind the major groove of the DNA. Each zinc finger has two anti-parallel β sheets folded in on an α helix. Inside the fingers, two histidines within the helix and two cysteines within the β sheets are important for chelating the zinc atom (28). The carboxy-terminal zinc fingers are required for pericentromeric targeting because IKAROS dimerization is essential for DNA-binding (19). However, there is also some evidence of *in vivo* IKAROS multimerization which helps to reconcile the binding of IKAROS to both, target genes and pericentromeric repeats (30). IKAROS-null (*IKZF1*null/null) mice are characterized by a lack of hematopoietic stem cells (HSCs), the absence of B cells and their progenitors, and they are prone to the development of T-cell leukemia/lymphoma with high penetrance (18, 31, 32).

In humans, a germline mutation in *IKZF1* was first described in an infant with pancytopenia and loss of B cells (33). More recently, autosomal dominant heterozygous loss of-function germline mutations in *IKZF1* associated with CVID-Like phenotype (hypogammaglobinemia with autoimmune manifestations) have been reported in 42 patients of 15 non-related families. These mutations in *IKZF1* impair the DNA binding of IKAROS to its target sequence and cause an immunodeficiency syndrome predominantly characterized by an antibody insufficiency (16, 34–37). Apart from progressive loss of B cells and serum immunoglobulins, hematopoietic malignancies such as predisposition to B cell precursor acute lymphoblastic leukemia (16) and subsequent T-cell leukemia were also reported in four patients in these studies (37, 38). Additionally, a *de novo* heterozygous germline mutation in *IKZF1* has been identified recently in 7 unrelated patients with an early-onset combined immunodeficiency. The patients were characterized by defects in innate and adaptive immune system, including low B cell numbers and impaired function of neutrophils, eosinophils, and myeloid dendritic cells, as well as T cell and monocyte. One patient in this cohort was reported to develop a T cell ALL (38).

Here, we characterize eleven patients with heterozygous *IKZF1* variants from eight different families with recurrent bacterial infections of the respiratory tract, antibody isotype deficiencies involving IgM, IgG, and IgA, and autoimmune manifestations, with an autosomal dominant mode of inheritance. In addition, we describe two siblings with inflammatory bowel disease (IBD) carrying an *IKZF1* variant. Our study shows that mutations affecting the DNA binding domain of IKAROS can impair the interaction with the target DNA sequence thereby preventing heterochromatin-pericentromeric localization (HC-PC). Our results also showed that the pericentromeric localization of IKAROS is impaired by the overexpression of the truncated variant Lys286^*^ in NIH3T3 cells. Although still able to bind to the target DNA as a dimer, complexes of wildtype and the Met494Val variant were unable to form oligomers of IKAROS with the target DNA sequence.

## Results

### Heterozygous Variants in *IKZF1* in a Cohort of Primary Immunodeficiency Patients With Variable Clinical Manifestations

**Figure 1:**
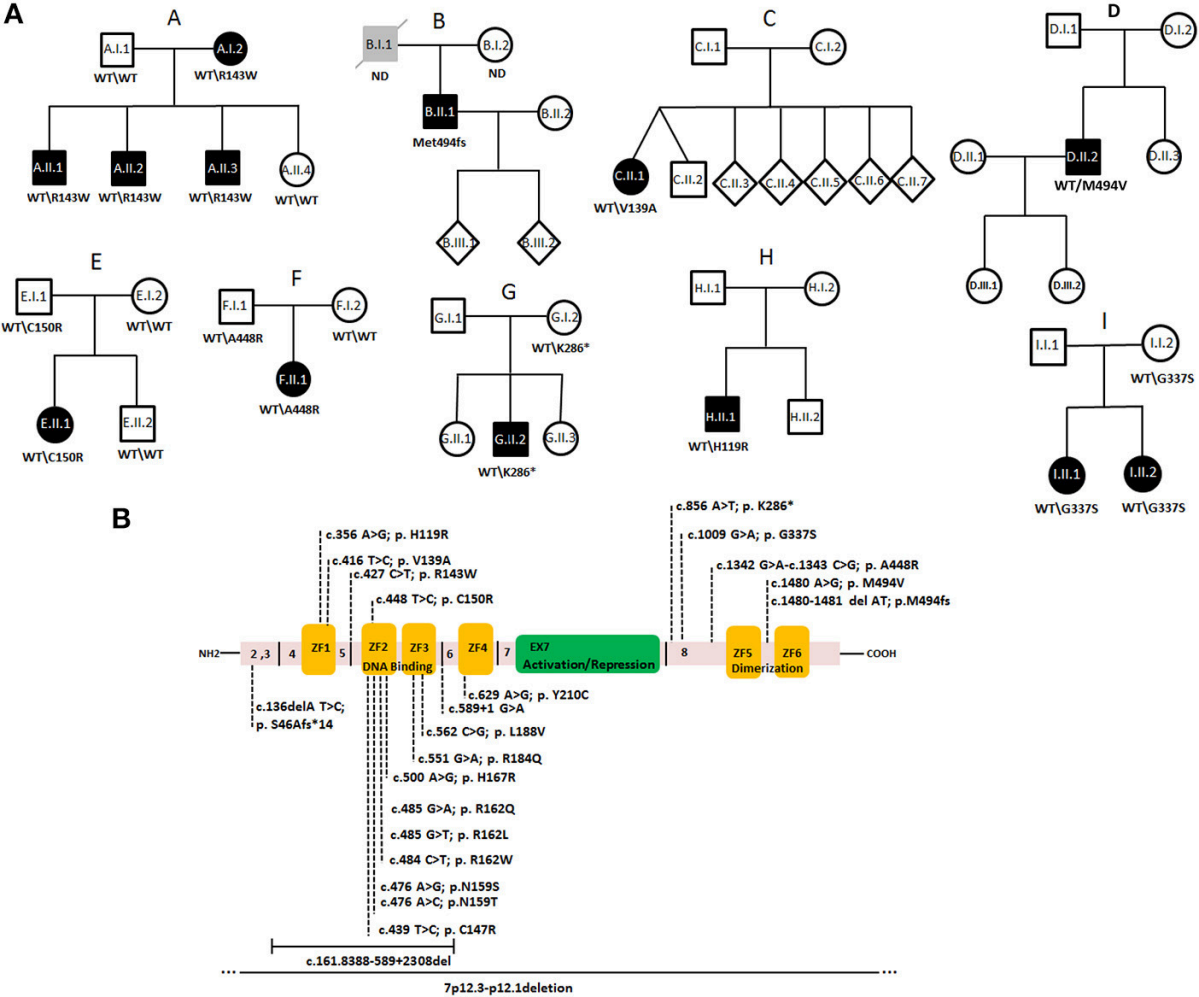
Autosomal dominant inheritance of IKAROS heterozygous mutations in CVID families of a cohort of 650 individuals. **(A)** Pedigrees of families A–I. Circles, female; squares, male; black filled symbols, affected individual; gray filled symbols; individual with hypogammaglobulinemia without CVID-related symptoms, open symbol; unaffected; slash, diseased. **(B)** Schematic presentation of the structure of human IKAROS. The N-terminal DNA binding domain composed of four zinc finger motifs (yellow boxes, ZF1–ZF4), the central activation/repression domain (green) and the C-terminal dimerization domain (ZF5–ZF6). Exon borders and amino acid positions are indicated. Dotted lines in the upper part indicate heterozygous variants identified in this study. Lower parts shows previously published *IKZF1* mutations (16, 34–38).

Genetic analysis including whole exome and targeted gene panel sequencing was performed in a cohort of 650 individuals with primary immunodeficiencies and resulted in the identification of one frameshift mutation, one non-sense mutation, and seven missense variants in *IKZF1* in nine unrelated families (Figures 1A,B).

In Family A, we detected a heterozygous missense variant (NC_000007.14, g.50450243C>T, c.427C>T, p.Arg143Trp NM_006060.6, rs757521297) in exon 4 of *IKZF1*, which segregated with the clinical phenotype (Figures 1A,B). The identified variant affects the linker region between ZF1 and ZF2 and it was predicted to be disease causing, based on *in silico* analysis using Polyphen 2 and SIFT. All affected family members were previously diagnosed with CVID, as they had hypogammaglobinemia associated with recurrent bacterial respiratory tract infections. Other clinical manifestations were variable and are displayed in Table 1.

**Table 1:** Clinical manifestations and laboratory findings of patients carrying *IKZF1* variants.

	**A.I.2**	**A.II.1**	**A.II.2**	**A.II.3**	**B.II.1**	**C.II.1**	**D.II.2**	**E.II.1**	**F.I.1**	**G.II.2**	**H.II.1**	**I.II.1**	**I.II.2**	**Normal**
Age	53	19	29	23	45	41	44	36	32	16	NA^*^	10	15 months	
Age of onset	29	19	24	18	37	20	19	17	28	16	NA	2nd week of life	2nd week of life	
Sinopulmonary infections	Yes	Yes	Yes	Yes	No	Yes	Yes	Yes	No	Yes	NA	NA	NA	
Autoimmunity	Hashinoto thyroiditis/ urticaria	Scaling of the skin	No	No	ANCA-negative vasculitis	No	Autoimmune cytopenia/ IT	Seronegative arthritis and tenosynovitis	No	Yes	PAN	Antigliadin IgA and tissue transaminase IgA positivity	NA	
ALL	No	No	No	No	No	No	No	No	No	Yes	No	No	No	
Splenomegaly	Yes	No	No	Yes	No	No	Yes	No	Yes	Yes	No	NA	NA	
Other clinical manifestations	Chronic gastritis	None	Otitis	Otitis	None	None	Hemolytic anemia-CNS lymphoma	Recurrent diarrhea/ malabsorption/ Asthma	EBV positive, HPV genital infection	Chronic recurrent diarrhea, sever cellulitis	Had only on kidney	Chronic diarrhea	Chronic diarrhea	
CD19 B cells [μl]	20	106	29	72	57	371	95	0	–	100	NA	NA	NA	100–500
IgM^++^CD38 transitional [%]	8.47	2.8	25.72	17	0.1	1	12.02	NA	3.04	NA	NA	NA	NA	0.5–4.4
IgA^+^CD27 B cells [%]	0.16	1.3	3.8	0.49	4.1	0.02	NA	NA	NA	NA	NA	NA	NA	2.8–10.9
IgG^+^CD27 B cells [%]	0.12	2.3	1.6	0.39	4	0	0.22	NA	0.82	NA	NA	NA	NA	2.5–20.3
IgG [g/l]	1.13	3.24	5.5	3.4	4.38	3.15	6.95	0.32	15.6	<0.1	NA	NA	NA	7–16
IgM [g/l]	0.28	1.4	0.2	0.23	0.54	1.61	0.14	0.11	2.52	0.45	NA	NA	NA	0.4–2.3
IgA [g/l]	0.28	0.55	1.2	1.18	0.24	0.06	4.92	0.05	2.03	<0.1	NA	NA	NA	0.7–4
Plasma blasts from B cells[%]	0.8	2.3	4.7	0.3	0.06	0	0.04	NA	0.02	NA	NA	NA	NA	0.4–3.6
CD3^+^CD8^+^ T cells [μl]	566	310	699	402	192	1083	91	741	269	NA	NA	NA	NA	200–900
CD4^+^/CD8^+^ T cells	1.09	2.8	1.6	1.8	0.89	1	2.69	0.7	1.24	NA	NA	NA	NA	1–3.6
CD45RO^+^ from CD3^+^CD4^+^Tcells[%]	8.27	49.7	59.4	39.1	36.3	87	95.7	NA	NA	18	NA	NA	NA	35–73

* *NA, Not available*.

A heterozygous frame-shift variant (g.50468244delAT, c.1480_1481delAT, p.Met494fs^*^86) was detected in patient B.II.1. Despite having low levels of IgG and IgA, this patient was not reported to have respiratory tract infections, but was diagnosed with an ANCA-negative vasculitis with pulmonary involvement. His father was reported to have IgA and IgM deficiency, but no related symptoms were reported and he died from alcohol-induced liver cirrhosis.

A unique missense variant (transcript 15, ENST00000413698.5, NM_001291845, g.50435959, c.416T>C, p.Val139Ala) affecting the first zinc finger of IKAROS was identified in the CVID-affected individual of Family C who had sinusitis, repeated severe pneumonia, and low levels of IgG and IgA.

In patient D.II.1, who developed interstitial pulmonary disease and autoimmune cytopenia, a heterozygous missense variant (g.50468245A>G, c.1480A>G, p.Met494Val) occurred at the identical amino acid position which was affected by a frame-shift variant in Family B. The daughter of this patient was diagnosed with atopic dermatitis and his cousin was reported to have autoimmune thrombocytopenic purpura (ITP).

In Family E, a heterozygous missense change (g.50450264T>C, c.448T>C; p.Cys150Arg) was identified in patient E.II.1 who had chronic rhinosinusitis and frequent respiratory infections. Of note, his unaffected father also carried the variant.

In Family F, the affected individual had hepatosplenomegaly and recurrent fever of unknown origin. He and his healthy father both carried a short insertion/deletion variant (g.50468107-50468108insdelAG, c.1342-1343insdelAG, rs765655969 and rs750934235) leading to a single amino acid substitution p.Ala448Arg.

A non-sense mutation (g.50467621A>T, c.856A>T, p.Lys286^*^) was identified in the patient of Family G (G.II.1) with early onset CVID and B cell acute lymphoblastic leukemia (B-ALL). The same mutation was found in his asymptomatic mother.

In Family H, a missense variant (g.50435899, c.356A>G, p.His119Arg, rs117111762) was detected in a patient with polyarthritis nodosa (PAN).

A missense variant (g.50467774G>A c.1009G>A, p.Gly337Ser, rs148169768) was identified in patients I.II.1 and I.II.2, both diagnosed with IBD after developing chronic diarrhea and malabsorption (Table 1). The variant predicted a single amino acid change in the linker region between the activation/repression site and the dimerization domain of IKAROS.

Sanger sequencing confirmed the above variants in all available samples of each pedigree (Figure S1). Among the identified variants, the non-sense mutation p.Lys286^*^ in Family G was assumed to cause haploinsufficiency of IKAROS due to expression of a severely truncated non-functional protein which probably undergoes rapid decay. By contrast, none of the missense variants has previously been studied or has been described as disease-causing and therefore functional consequences were unpredictable. To study the impact of the missense variants on IKAROS function, we overexpressed the mutant proteins *in vitro* and evaluated expression and nuclear translocation by western blotting, the DNA binding properties by electrophoretic mobility shift assay (EMSA), and the sub-nuclear localization in pericentromeric-heterochromatin (PC-HC) by microscopic analysis. As all variants were heterozygous, DNA-binding and sub-nuclear localization was also assessed upon co-transfection with equal amounts of wildtype and mutant IKAROS.

### IKAROS Missense Variants Have Expression Levels Similar to the Wildtype Protein in Transfected Cells

To examine the stability of the proteins resulting from the mutations in *IKZF1* and their ability to enter the nucleus, we analyzed the expression of wildtype and mutant IKAROS in transiently transfected HEK293T cells by Western blotting of nuclear extracts. All ectopically expressed IKAROS missense variants had the expected molecular weight of 63 kDa, as reported in previous studies (35), and protein levels were comparable to the wildtype control (Figures S2A,B). Thus, none of the identified variants affected the expression or nuclear localization *in vitro*. Since the epitope detected by the monoclonal antibody was not preserved in the p.Lys286^*^ non-sense variant, we used an N-terminally directed antibody to analyze the expression of the truncated protein. A single band with the expected size (31 kDa) was observed, confirming that the overexpressed truncated protein is stable and can enter the nucleus in transfected cells. Thus, in spite of being predicted to be haploinsufficient by *in silico* analysis, the non-sense mutation may not prevent the expression of the protein and the truncated protein may be expressed at the predicted size and could be translocated to the nucleus. In cells transfected with wildtype IKAROS, three main bands with the expected size of the full-length protein (55–80 kDa) and an additional smaller band of 30 kD were detected (Figure S2C). The fourth band (which is distinct from the truncated p.Lys286^*^ variant as confirmed by co-transfection and only detectable with the N-terminal antibody) might be due to protein fragments originating from the ectopic overexpression of the wildtype IKAROS.

### Stability and Nuclear Localization of Wildtype IKAROS Is Unaffected by Co-expression of Missense Variants

Since all variants identified in this study were heterozygous, we questioned whether assembly of an IKAROS wildtype monomer with a mutant counterpart can change expression, stability, and/or nuclear localization of the mixed dimers. Western blotting with nuclear extracts from HEK293T cells transiently co-transfected with equal amounts of wildtype and mutant IKAROS showed a single band with the expected size (63 kDa for missense variants and wildtype IKAROS; 31 kDa for the p.Lys286^*^ truncation) in all samples analyzed and no increased ubiquitination and/or degradation or any other sign of dimer-decay was detected (Figures S2A–C). The results indicated that the identified missense variants do not affect the protein stability and subcellular localization of IKAROS. Moreover, dimer-decaying effects were not observed.

### Variants Affecting the DNA Binding Domain of IKAROS Impaired Its Binding Ability to the Target Sequence

**Figure 2:**
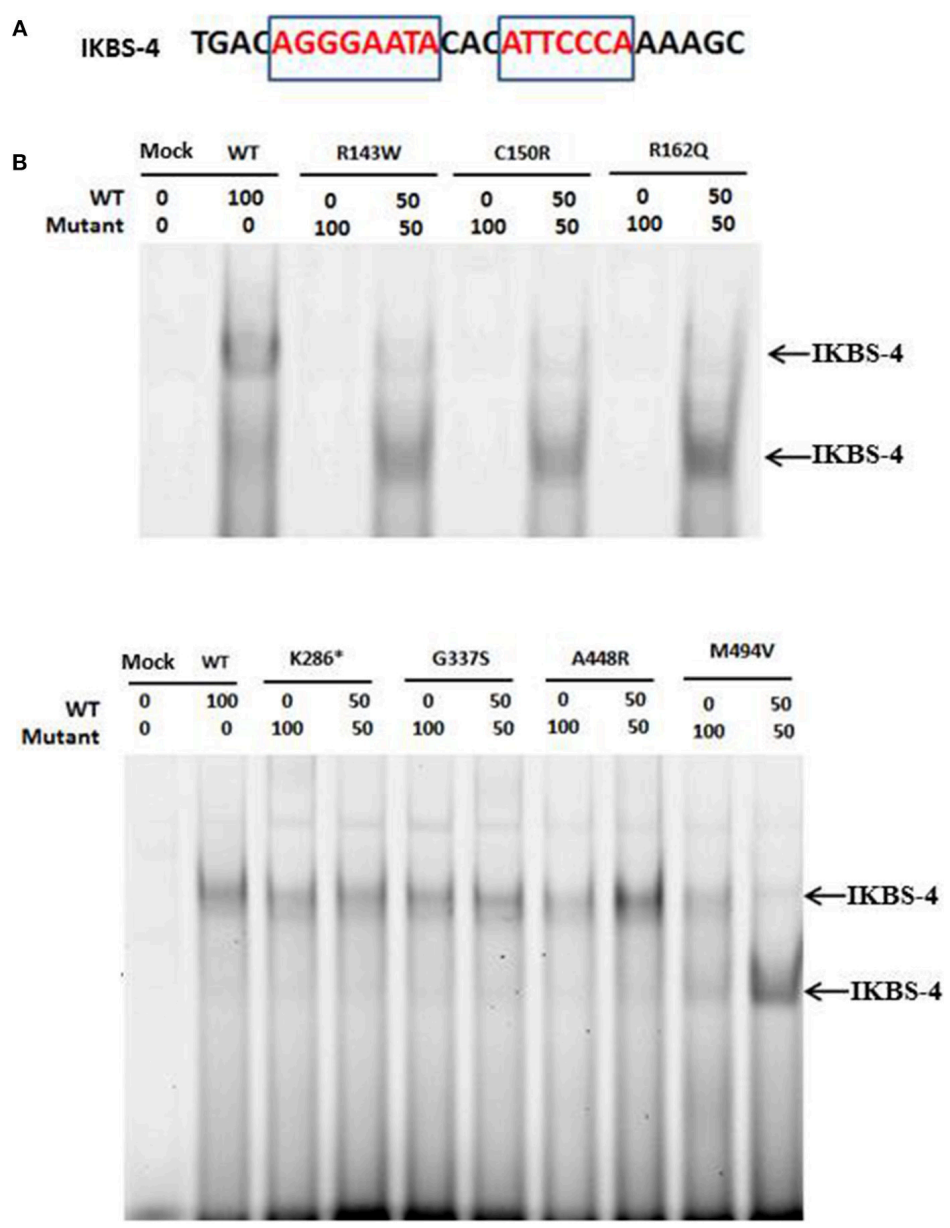
**(A)** The sequence, used for the EMSA experiments is shown. Motifs that resemble the IKAROS consensus are shown in red in the rectangle. **(B)** Mutations affecting the DNA binding domain of IKAROS, preventing its binding to its target sequence. A specific gel-shift band, indicating binding of IKAROS multimers to the IKBS-4 DNA probe, was observed in the nuclear extract of HEK293T cells expressing the wildtype IKAROS. This band was absent in cells expressing the IKAROS variants Arg143Trp, Cys150Arg, and Arg162Gln and was weaker in the nuclear extracts of cells co-transfected with equal amounts of wildtype and one of the three mutant forms. A smaller band was also detected in the co-transfected samples, compared to the wildtype ones which can indicate the DNA-bound dimers of IKAROS. The DNA binding activity remained unaffected when the mutant variants Lys286*, Gly337Ser, Ala448Arg, and Met494Val were expressed.

Since two of the identified variants (p.Arg143Trp and p.Cys150Arg) studied here affected the DNA binding domain of IKAROS and Cys150 is one of the cysteines of the C2H2 domains responsible for Zn^2^^+^ coordination, we asked whether these mutant proteins retain their ability to bind to the target DNA sequence. We therefore transiently expressed either wildtype or mutant IKAROS in HEK293T cells and then analyzed the intrinsic DNA-binding ability of wildtype and mutant IKAROS in nuclear extracts by EMSA, using an infrared labeled synthetic oligonucleotide probe (IKBS-4) which contains a high-affinity IKAROS binding site. As expected, a specific gel-shifted band was observed in the wildtype IKAROS-expressing HEK293T cells, indicating binding to the IKBS-4 DNA probe (Figure 2A). This band was absent in cells expressing the IKAROS variants p.Arg143Trp or p.Cys150Arg, both of which affect the DNA binding site of IKAROS and thus interfere with the DNA binding ability. DNA binding was also not detectable with the p.Arg162Gln mutant (rs770551610), which was included as negative control, consistent with a previous report (16). From these results we concluded that either dimers composed of two mutant p.Arg143Trp or p.Cys150Arg monomers are unable to bind efficiently to DNA or dimerization was not stable enough to interact with the targeted sequence. In contrast to mutations in the DNA binding domain, the DNA binding activity remained unaffected when the mutant variants p.Lys286^*^, p.Gly337Ser and p.Ala448Arg and p.Met494Val were expressed. The observation that variants in the linker region showed no detectable difference when compared to wildtype IKAROS indicate that the variants affecting the linker region and also the region between ZF5 and ZF6 in the dimerization domain did not affect the DNA binding ability of IKAROS.

### The DNA Binding Ability of Wildtype IKAROS Remains Unaffected in the Presence of Mutant Variants With DNA Binding Defects

As previously explained, all identified variants in this study were heterozygous. Thus, in order to find out whether the interaction of wildtype and each of the variant forms can interrupt the DNA binding of wildtype IKAROS to the target sequence of IKBS-4, we performed EMSA with the nuclear extract of cells, co-transfected with equal amounts of wildtype and each mutant form. In nuclear extracts of cells co-transfected with 50% of wildtype and 50% of p.Arg143Trp, p.Cys150Arg, or p.Arg162Gln, DNA binding was observed, but was weaker compared to the cells overexpressing 100% the wildtype construct. Therefore, variants in the DNA binding domain did not abolish the DNA binding ability of mixed wildtype/mutant IKAROS to the target sequence. However, the gel-shifted band in the co-transfected samples had a smaller size, compared to the wildtype sample (Figure 2B), indicating that although mixed wildtype/mutant dimers can bind to DNA, they are unable to form multimers. Oligomerization of the protein resulting from the co-transfection of wildtype IKAROS with p.Met494Val variant, appeared to be impaired while the dimers of IKAROS were still able to form some complexes with the target sequence. Upon co-transfection of wildtype IKAROS with the mutant variants p.Lys286^*^, p.Gly337Ser, p.Ala448Arg, the DNA binding ability as well as formation of high-molecular weight multimers remained unchanged compared to the single transfection of wildtype IKAROS alone.

### Variants Affecting the DNA Binding Region of IKAROS Cause Diffuse Nuclear Localization

**Figure 3:**
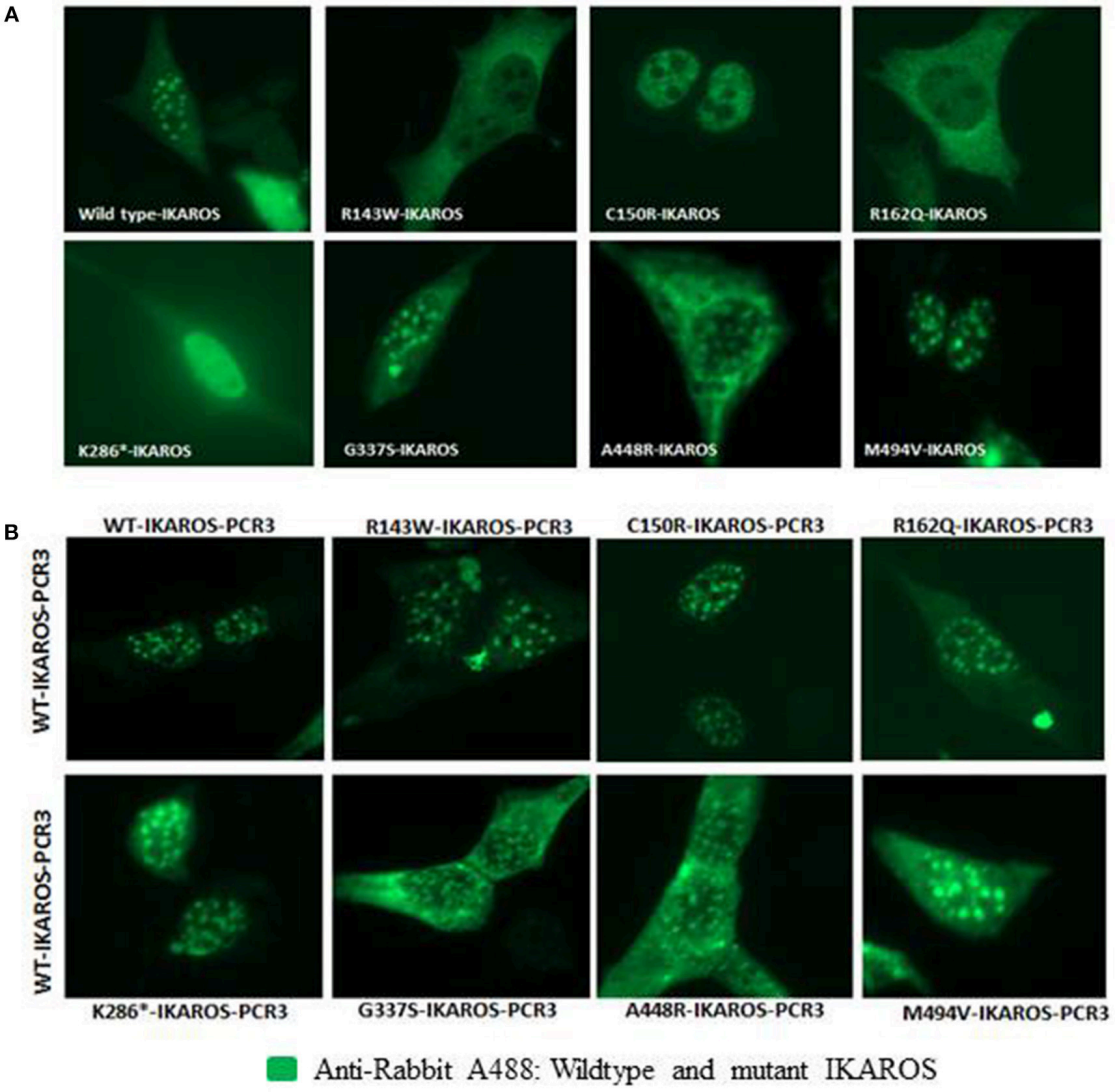
IKAROS subnuclear and pericentromeric-heterochromatin localization. **(A)** NIH3T3 cells, transiently transfected with the pCR3 vector expressing wildtype or mutant IKAROS were incubated with rabbit-anti-human-IKAROS antibody and N-terminus anti-human IKAROS antibody and stained with goat anti-rabbit Alexa fluor 488. Cells overexpressing the wildtype IKAROS, showed the punctate staining pattern, observed in green. In contrast, a diffuse nuclear staining was observed in NIH3T3 cells overexpressing the Arg143Trp, Cys150Arg, Arg162Gln, and Lys286* mutant forms. **(B)** The DNA binding ability of wildtype IKAROS was not inhibited by the presence of a mutant variant. NIH3T3 cells were co-transfected with equal amounts of wild-type and each mutant pCR3 expression vectors and incubated and stained with the above-mentioned antibodies. In NIH3T3 cells co-transfected with equal amounts of wild-type and each mutant forms, the punctate nuclear staining pattern were obtained in all samples.

**Figure 4:**
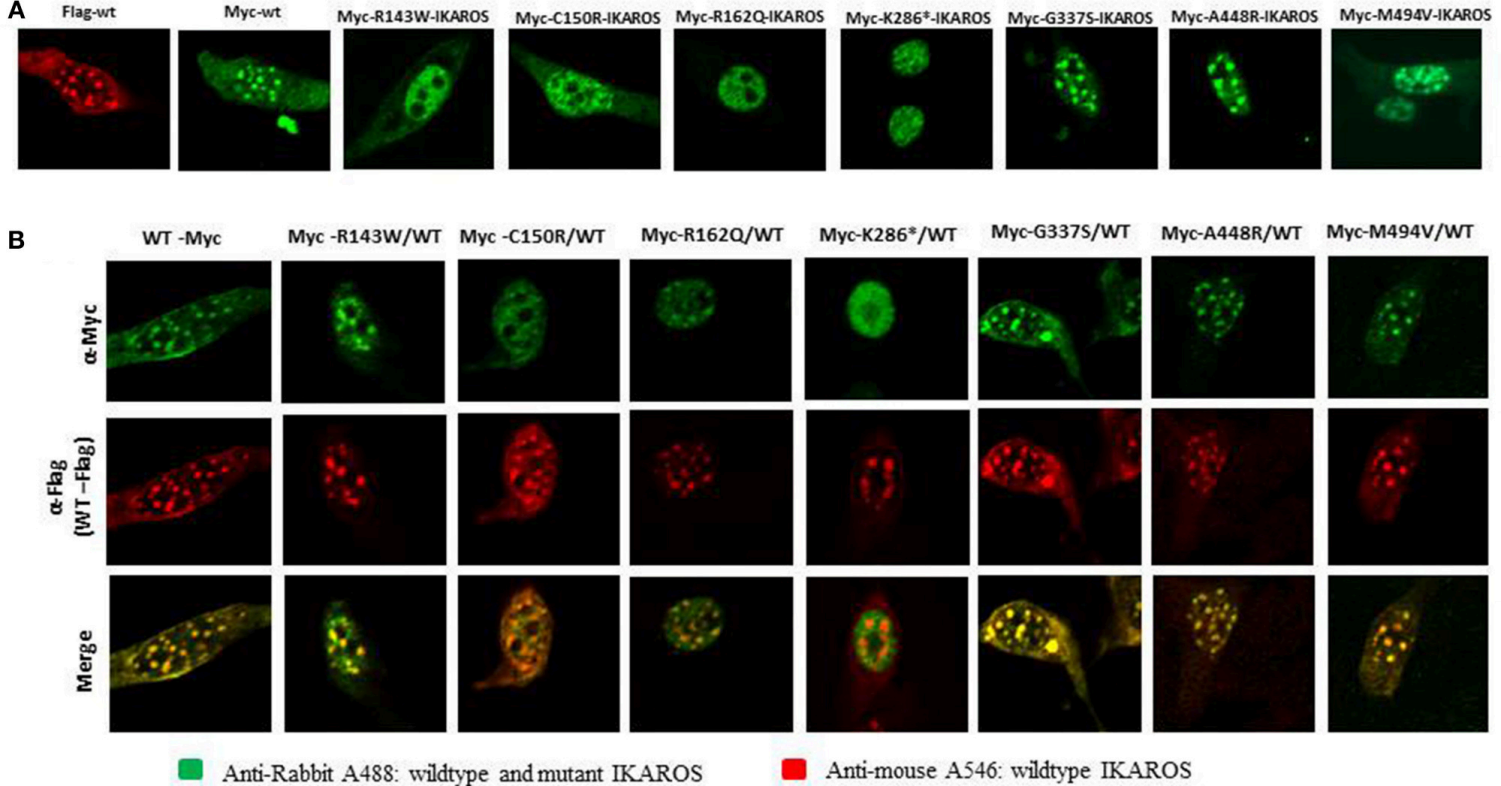
The results of confocal microscopy with epitope-tagged constructs were consistent with the results of Immunofluorescent microscopy. **(A)** NIH3T3 cells, transiently overexpressing epitope-myc/FLAG-tagged wildtype IKAROS and myc-tagged mutant forms were incubated with monoclonal rabbit anti myc-tag-antibody and mouse monoclonal anti-FLAG M2 antibody and stained with goat anti-rabbit Alexa fluor 488 (green) and goat anti-mouse Alexa 546 (red). In NIH3T3 cells transfected with epitope-myc/FLAG-tagged wildtype IKAROS the speckled nuclear localization was observed in contrast to a diffuse nuclear staining, observed in NIH3T3 cells expressing the myc-tagged Arg143Trp, Cys150Arg, Arg162Gln, and Lys286* mutant forms. **(B)** Wildtype components were dominant to form IKAROS complexes in PC-HC sites. NIH3T3 cells were co-transfected with 50% of myc/FLAG-tagged wildtype IKAROS and 50% of myc-tagged, expressing each mutant form. The diffuse nuclear localization of the mutant forms was significantly reduced in NIH3T3 cells co-transfected with equal amounts of the FLAG-tagged wildtype IKAROS together with the myc-tagged Arg143Trp, Cys150Arg, and Arg162Gln. Accumulation of FLAG-tagged wildtype proteins, observed as a punctuated nuclear staining pattern in the co-transfected samples, showed the dominance of wildtype components to form IKAROS complexes in PC-HC sites.

Localization of IKAROS to the pericentromeric-heterochromatin is crucial for its normal function in chromatin remodeling and regulation of its target gene expression (19). Thus, we performed immunofluorescence staining and confocal microscopy with transiently transfected murine fibroblasts (NIH3T3) to investigate the sub-nuclear localization of wildtype and mutant IKAROS. As expected, cells transfected with an expression vector for wildtype IKAROS alone had the punctate staining pattern that is characteristic of pericentromeric-heterochromatin binding and localization of IKAROS. The punctate staining pattern was also observed in cells transiently expressing the p.Gly337Ser, the p.Ala448Arg, or the p.Met494Val variants (Figure 3A). In contrast, a diffuse nuclear staining was observed in NIH3T3 cells expressing the p.Arg143Trp, p.Cys150Arg, p.Arg162Gln, and p.Lys286^*^ mutant forms, indicating that these variants lost their ability to bind DNA at the pericentromeric regions. We also analyzed the sub-nuclear (co)-localization of epitope-tagged wildtype and mutant IKAROS by confocal microscopy of transfected NIH3T3 fibroblasts. As expected, a punctuated nuclear staining pattern was observed in cells transfected with either FLAG- or myc-tagged wildtype IKAROS. Similarly, myc-tagged p.Gly337Ser, p.Ala448Arg, and p.Met494Val also showed a speckled nuclear pattern indistinguishable from the wildtype staining. In contrast, all of the myc-tagged p.Arg143Trp, p.Cys150Arg, p.Arg162Gln, and p.Lys286^*^ variants showed a miss-localized diffuse nuclear staining, indicating an impaired DNA binding ability (Figure 4A). In summary, missense mutations affecting the DNA binding domain of IKAROS abolished both, DNA-binding and localization to pericentromeric-heterochromatin regions.

### The DNA Binding Ability of Wildtype IKAROS Remains Unaffected Upon Co-expression of Mutant Variants

Since all identified variants in this study were heterozygous and the mutant IKAROS changed its characteristics when co-expressed together with the wildtype counterpart in EMSA, we investigated the interaction between wildtype and mutant IKAROS.

We analyzed NIH3T3 cells co-transfected with equal amounts of expression vectors for wildtype and each mutant forms. The punctate nuclear staining pattern was obtained in all the samples (Figure 3B). These results indicate that the DNA binding ability of wildtype IKAROS is not inhibited by the presence of a mutant variant and that the dimers composed of one wildtype and one mutant monomer are directed to the wildtype localization because wildtype is dominant over mutant.

In co-transfected cells, 25% of the dimers are expected to be wildtype/wildtype dimers, 50% hybrid dimers and 25% mutant/mutant dimers. With co-transfected FLAG-tagged wildtype IKAROS and myc-tagged variant, the mutations p.Gly337Ser, p.Ala448Arg, and p.Met494Val, showed an invariant speckled nuclear pattern, indicating that neither of these variants affected its sub-nuclear localization. In contrast, upon co-transfection of equal amounts of the FLAG-tagged wildtype IKAROS together with the myc-tagged p.Arg143Trp, p.Cys150Arg, and p.Arg162Gln, the diffuse nuclear localization of the mutant forms was markedly reduced, but accumulated at the normal localization of the FLAG-tagged wildtype proteins (Figure 4B). This observation suggests that besides wildtype/wildtype dimers (speckled nuclear localization) both mutant/mutant dimers (diffuse nuclear localization) and wildtype/mutant dimers (speckled nuclear localization) can assemble, with a clear dominance of the wildtype component. Thus, p.Arg143Trp, p.Cys150Arg, and p.Arg162Gln do not abolish nuclear entry but prohibit pericentromeric-heterochromatic localization with a reversion of the localization defect, once a mutant monomer interacts with a wildtype counterpart. However, the localization defect of the myc-tagged p.Lys286^*^ mutant, was not reversed with co-expression of wildtype IKAROS, indicating that the severely truncated p.Lys286^*^ variant lost its ability to bind HC-PC sites, consistent with the loss of its dimerization domain. The localization of the co-transfected wildtype IKAROS remained unaffected in these cells.

In summary, our results suggest that the pericentromeric-heterochromatin localization of wildtype IKAROS cannot be inhibited by the presence of IKAROS harboring mutations affecting the DNA binding domain. Moreover, the accumulation of FLAG-tagged wildtype proteins, which was observed as a punctuated nuclear staining pattern in the co-transfected samples, showed the dominance of wildtype monomers to form multimeric IKAROS complexes within pericentromeric-heterochromatin distribution.

### Possible Contribution of a Variant in *TNFSF10* (TRAIL) to the CVID Phenotype in Family A

Analysis of all variants segregating with the affected state in Family A, revealed one additional heterozygous missense variant (g.172241096, c.79G>T, p.Gln27Lys [Q27K]) in *TNFSFS10* (*TRAIL*), which perfectly segregated with the phenotype (Figures S3A,B). This variant was confirmed by Sanger sequencing (Figure S3C).

In order to reveal the functional relevance of the identified variant in *TRAIL*, we ectopically overexpressed the wildtype and the identified variant of TRAIL in HEK293T cells. Western blotting, performed in whole cell lysates of HEK293T cells overexpressing the wildtype and the p.Gln27Lys TRAIL showed that both wildtype and p.Gln27Lys TRAIL were expressed at the expected size of 28–31 kDa but the glycosylation pattern and the cleavage of TRAIL were impaired by the p.Gln27Lys variant which was affected the transmembrane domain of TRAIL (Figure S3D). Since the main known function of TRAIL is the induction of apoptosis upon binding to its death receptors. Thus, we questioned whether the apoptotic function of TRAIL can be affected by the identified variant. TRAIL-induced apoptosis was delayed in Jurkat cells, co-cultured with HEK293T cells expressing p.Gln27Lys form (Figure S3E). Additionally, the percentage of viable IgM^+^BJAB cells was significantly higher in the co-culture model of Gln27Lys TRAIL (Figures S3F,G). The surface localization of TRAIL showed a significant reduction in HEK293T cells overexpressing p.Gln27Lys TRAIL in the co-culture system (Figures S3H,I). To evaluate the effects of the identified variants in TRAIL-induced NFκB activation, the NFκB dual luciferase reporter assay was performed by using HEK293T cells, transiently transfected with a serial dilution (from 1.6 to 12.8 ng) of wildtype and p.Gln27Lys TRAIL constructs and the results showed that the p.Gln27Lys mutation in TRAIL causes the failure of the NFκB activation pathway in HEK293T cells (Figures S3J,K). The results of expression analysis by flow cytometry revealed that the p.Gln27Lys affected the membrane localization of TRAIL and led to the reduced surface expression of the protein (Figures S3L–N). By contrast, the intracellular staining of cells expressing the wildtype and p.Gln27Lys mutant form, showed no significant difference indicating that the surface translocation of TRAIL was affected by p.Gln27Lys mutation and the protein could be mainly accumulated in the endoplasmic reticulum (ER) of the expressing cells (Figures S3O,P). This result was confirmed by confocal microscopy analysis where pEGFP-C1-TRAIL-Gln27Lys construct showed less plasma membrane localization but a diffused ER distribution (Figure S3Q). Taken together, our functional evaluation indicated the failure of apoptosis and NFκB activation pathway in cells expressing Gln27Lys variant which could be the consequences of impaired membrane localization of the protein.

## Materials and Methods

### Study Participants

In this study, 11 individuals, previously diagnosed with CVID, based on findings consistent with this phenotype and two patients with chronic diarrhea, categorized as IBD were recruited from our primary immunodeficiency (PID) cohort which included 650 patients in total.

### Genetic Analysis and Bioinformatics

The genomics DNA of all the patients was extracted from whole blood samples by Qiagen extraction kit according to the manufacturer's protocol. Exome sequencing was performed for all the affected individuals of Family A, Family B, Family C and Family D, and Family E. The TruSeq Exome Enrichment Kit (Illumina) was used to enrich the samples. Reads of 2 × 100 bp paired-end were sequenced for one quarter lane per sample on the Illumina HiSeq2000. The FASTQ files, containing raw sequencing reads, were mapped against the human reference genome build UCSC hg19 using BoWTie 2 v2.2.3 (39), reordered, sorted, and converted to BAM format. The polymerase chain reaction (PCR) duplicates were removed with Picard v1.115 (http://broadinstitute.github.io/picard/). Local realignment around InDels, base quality score recalibration and variant quality score recalibration as well as variant calling were performed with the GATK v3.1 (40) based on their best practice recommendations.

Genetic variation data were saved in VCF files and handled using VCF tools program package (41). The common variants with the allele frequencies >0.01 in dbSNP database or in ExAC (Exome Aggregation Consortium database, Broad Institute) were then excluded from the data. The genes related to the immune system were designated using IRIS list (42) and GO list. For generating the GO list, genes annotated in Gene Ontology (43) were composed directly and indirectly with the term “immune system process” (GO:0002376) defined as “Any process involved in the development or functioning of the immune system, an organismal system for calibrated responses to potential internal or invasive threats.” http://amigo.geneontology.org/amigo/term/GO:0002376.

Sequencing data of the families (Family F, G, H, and I) in this study were generated by targeted panel resequencing. The target enrichment was performed by using a HaloPlex Target Enrichment System for Illumina Sequencing (Agilent Waldbronn Germany). A customized gene panel of 120 candidate genes was designed using Agilent's web-based SureDesign application. Briefly, the DNA samples were first, digested and later hybridizing the restriction fragments, capturing the target DNA, closing the circular fragments via the ligation reaction, and the purification of the amplified target libraries were performed according to the manufacturer's instructions of the Agilent's user manual. Samples were then pooled in equimolar amounts for multiplexed sequencing on a MiSeq system (Illumina- Eindhoven; The Netherlands) and were prepared for sequencing using Illumina version 2 reagent kits, based on the protocol. The libraries were denatured in NaOH and diluted to a final concentration of 8–12 pM. The samples were loaded in to the MiSeq system and the system was started according to the instructions of the user guide. Finally the Agile's SureCall software was used for data analysis. All the identified variants were confirmed by Sanger sequencing. The coding genomic regions of *IKZF1* were amplified from genomic DNA, by standard PCR. PCR primers were used for Sanger sequencing according to standard techniques. Primer sequences are available upon request from the corresponding author.

### Generation of IKAROS cDNA Expression Vectors

A non-tagged, IKAROS cDNA was cloned by RT-PCR into the mammalian expression vector pCR3 (kindly provided by Prof. Pascal Schneider-University of Lausanne) to generate pCR3-IKAROS-wildtype. Identified mutations of *IKZF1* were introduced in to the wildtype-IKAROS-pCR3 via site-directed mutagenesis. These constructs were used to analyze the pericentromeric-heterochromation (PC-HC) localization by indirect immunofluorescence microscopy and to evaluate the DNA binding ability by EMSA. To distinguish between wild type and mutant IKAROS, C-terminal FLAG, and myc-tags were introduced into the wildtype and mutant IKAROS constructs, respectively, by PCR using appropriate reverse primers (see primers sequences in Table S1). Two of the identified sequence variants (p.Val139Ala and p.His119Arg) were not included in our analyses since they only occur in isoform 15 (NM_001291845), which lacks the N-terminal zinc fingers motifs does probably not bind to DNA and thus has an unknown relevance. The shifted reading frame of variant p.Met494Valfs86^*^ extends into the 3' UTR and has not been pursued further in this study. The p.Arg162Gln (rs770551610) variant in *IKZF1* which was already identified as a deleterious mutation which can affect the DNA binding ability of IKAROS in previous studies (16) was used in this study as a control for the technical procedure.

### Fluorescence Microscopy and Confocal Imaging

NIH3T3 mouse fibroblasts were seeded onto collagenized coverslips and cultured in DMEM (1X) (Dulbecco's Modified Eagel Medium) supplemented GlutaMAX, with 10% Fetal calf serum (FCS) and 1% Penicillin/Streptomycin, in 24-well plates, 24 h prior to transfection and were allowed to attach and grow overnight. Cells were transfected with the indicated expression vectors (wildtype and mutant IKAROS cDNA in pCR3) using *jetPEI* transfection reagent (#101-01N, Polyplus, Illkirch, France), according to the manufacturer's instruction. For co-transfections equal amounts of wildtype and mutant vector DNA constructs were used. Forty 8 h post-transfection the cells were washed once in PBS-T (PBS with 10% Tween 20), fixed for 15 min in 4% paraformaldehyde and permeabilized with ice-cold methanol for 10 min at −20°C. Non-specific binding was blocked by incubating for 1 h in PBS-T supplemented with 2.5% skim milk powder and incubated overnight at 4°C with rabbit-anti-human-IKAROS antibody (# 9034, Cell Signaling, Frankfurt am Main, Germany). Cells over expressing the p.Lys286^*^ mutant were incubated with N-terminus rabbit anti-human IKAROS antibody (#ab229275, Cambridge, UK). Cells were washed with PBS-T three times and incubated with Alexa fluor-488 goat anti-rabbit secondary antibody (#A11070 Thermo Fisher Scientific, Darmstadt, Germany) for 2 h at room temperature. Nuclei were stained with Hoechst 33342 (#B2261 Sigma, Taufkirchen Germany). Cover slips were rinsed three times with PBS-T and mounted on glass slides using Mounting medium (#S3023 DAKO, Freiburg Germany). Images were collected with a Zeiss AxioObserver inverted microscope equipped with the 40 X/0.75 M 27 objectives (Carl Zeiss, Jena, Germany) and processed with the Zeiss ZEN-imaging software.

For co-localization studies, cells were either transfected with myc-tagged wildtype or mutant IKAROS constructs alone or co-transfected with pCR3-IKAROS-mutant-myc and pCR3-IKAROS-wildtype-FLAG. Samples were stained with monoclonal rabbit anti myc-tag-antibody (Clone 71D10; #2278 Cell Signaling, Frankfurt am Main Deutschland) and mouse monoclonal anti-FLAG M2 antibodies (#F1804 Sigma Aldrich, Taufkirchen Germany), respectively. Secondary antibodies were goat anti-rabbit Alexa fluor 488 (**#** A-11070 Thermo Fisher Scientific, Darmstadt Germany) and a goat anti-mouse Alexa 546 (**#** A-11030 Thermo Fisher, Darmstadt Germany). Images were collected using a Zeiss LSM710 confocal microscope equipped with a 63x/1.40 oil immersion DIC M27 objective and analyzed by ZEN microscope-software (ZIESS- Oberkochen-Deutschland).

### Preparation of Cytoplasmic and Nuclear Protein Extracts

Nuclear extracts were prepared from HEK293T cells transfected with wildtype or mutant pCR3-IKAROS expression vectors. Cells were harvested 48 h post-transfection, washed with PBS and transferred to micro-centrifuge tubes. Cell membranes were lysed by re-suspending the cell pellets in 60–70 μl of Buffer H (20 mM HEPES pH 7.9, 1.5 mM MgCl_2_, 10 mM KCl, 0.1% NP40; and freshly added 1 mM DTT 1 μl and 1X proteinase inhibitor cocktail) and supernatants containing cytoplasmic proteins were collected by centrifugation at 14,000 g for 5 min at 4°C. Nuclei were re-suspended in 60 μl nuclear extraction buffer D (20 mM HEPES pH 7.9, 1.5 mM MgCl_2_, 0.2 mM EDTA, 0.42 M KCl, 20% Glycerol, and 1 mM DTT and 1X proteinase inhibitor cocktail in the final volume) and agitated for 1 h on a laboratory shaker at 4°C. Supernatants contained the nuclear proteins were collected by 5 min centrifugation at 14,000 g and 4°C.

### Western Blotting-IKAROS

Proteins were separated by standard SDS-PAGE and transferred to PVDF membranes. The membranes were incubated with monoclonal rabbit anti-human IKAROS (#9034 Cell signaling, Frankfurt am Main Germany). This antibody bound the residues surrounding Arg439 and was not able to detect the truncated form of IKAROS, generated by the constructs expressing p.Lys286^*^. Thus, the expression of IKAROS in HEK293T cells expressing wildtype IKAROS and the mutant p.Lys286^*^ was analyzed by western blot with N-terminus anti-human IKAROS antibody (#ab229275, Cambridge, UK). Equal protein loading was assured by TATA box-binding protein (TBP) level, using Rabbit anti-TATA box binding protein (#8515 Cell Signaling, Frankfurt am Main Germany), and visualized by chemiluminescence using donkey anti-rabbit HRP-linked secondary antibodies (#7074S Cell Signaling, Frankfurt am Main Deutschland). The Fusion Cap advance acquisition (Vilber Lourmat-Eberhardzell-Germany) was used for image development and processing.

### Electrophoretic Mobility-Shift Assay

Buffer HGED (20 mM HEPES pH 7.9, 0.2 mM EDTA, 20%Glycerol, 1 mM DDT) was added to 10–12 μg of the nuclear extracts of transfected HEK293T cells, to the total volume of 12.5 μl.

The EMSA probe was obtained by annealing two DY682 Infra-red dye probes oligonucleotides (stock of 100 pMol), corresponding to the regulatory regions of IKAROS binding site-4 (IKBS-4).

Forward: 5′-TGACAGGGAATACACATTCCCAAAAGC-3′

Reverse: 5′-GCTTTTGGGAATGTGTATTCCCTGTCA-3′

Briefly, the annealing reaction was performed by adding 4 μl of each oligo (6 pmol) to 192 μl of the annealing buffer (Tris pH 7.5, 50 mM NaCl). The annealing reaction was 2 min at 95°C, followed by 1 min of gradual cooling down at 25°C and finally 4°C. The probes were pre-incubated with binding solution (1 μg/μl BSA, 1 μg/μl polydI:dC, 10 μM ZnCl_2_, in 12.5 μl H_2_O) for 15 min at room temperature. Samples were added to the binding solution and were incubated for 45 min at room temperature. DNA-protein complexes were separated on 6% acrylamide gels with 1 × TBE buffer (1.3 M Tris, 450 mM M Boric acid; 25 mM EDTA) at 80 V at room temperature. Images were obtained using an Odyssey CLx infrared scanner (Li-Cor- Nebraska USA).

### Generation of TRAIL cDNA Expression Vectors

A non-tagged, TRAIL cDNA was cloned by RT-PCR into the mammalian expression vector pCR3 to generate pCR3-TRAIL-wildtype. Identified mutation (c.79G>T) was introduced in to the wildtype-TRAIL-pCR3 construct via site-directed mutagenesis. Later, an N-terminal GFP-Tagged construct was also generated by pEGFP-C1 plasmid and the full-length wildtype cDNA of TRAIL and the one containing c.79G>T mutation by PCR using appropriate primers. This construct was used for confocal microscopy to analyze the membrane and cytoplasmic localization of the wildtype and the mutant form.

### Preparation of the Cell Lysate

Cytoplasmic extracts were prepared from HEK293T cells transfected with wildtype or p.Gln27Lys-pCR3-TRAIL expression vectors. The transfection was done by *jetPEI* transfection reagent (#101-01N, Polyplus, Illkirch France), according to the manufacturer's instruction. Cells were harvested 48 h post-transfection, washed with PBS and transferred to micro-centrifuge tubes and spun down at 400 g for 5 min at 4°C. The supernatant was discarded and cell membranes were lysed by re-suspending the cell pellets in RIPA buffer [0,1% SDS, 1:25 EDTA tablet from Roche [1 tablet, diluted in 1ml H2O], 1:100 Phosphate inhibitor cocktail 2 and 1:100 Phosphatase cocktail 3]. Cells were incubated on ice for 5 min and then spun down at 600 g for 10 min at 4°C. The supernatant which contained the cell lysate was transferred to a new micro tube.

### Western Blotting- TRAIL

Proteins were separated by standard SDS-PAGE and transferred to PVDF membranes. The membranes were incubated with monoclonal rabbit anti-human TRAIL (#3219 Cell signaling, Frankfurt am Main Germany) which bound the residues surrounding Lys60, for 2 h. Equal protein loading was tested by incubating the membrane with α-Tubulin expression level, using Rabbit anti-α-Tubulin (#ab108629 abcam, Cambridge, UK), and visualized by chemiluminescence using donkey anti-rabbit HRP-linked secondary antibodies (#7074S Cell Signaling, Frankfurt am Main Deutschland). The Fusion Cap advance acquisition (Vilber Lourmat- Eberhardzell- Germany) was used for image development and processing.

### Confocal Microscopy

6 × 10^5^ HEK293T cells were seeded onto the collagenized coverslips in DMEM (1X) (Dulbecco's Modified Eagel Meduim) + GlutaMAX with 10% FCS and 1% Penicillin/Streptomycin, in a 6-well plate, 24 h prior to transfection. Cells were transfected with the pEGFP-C1 vectors expressing wildtype and p.Gln27Lys mutant by *jetPEI* transfection reagent (#101-01N, Polyplus, Illkirch France). Forty 8 h post-transfection the cells were washed with PBS with 10% Tween 20, fixed with 4% paraformaldehyde, and permeabilized with ice-cold methanol. Cells were stained with wheat germ agglutinin conjugates/Alexa Flour 633 (#A22284-Invitrogen Darmstadt-Germany) according to manufacturer's instructions. Finally, cells were stained with Hoechst 33342 and embedded in fluorescence mounting medium. Confocal microscopy was performed on a Zeiss LSM710 confocal microscope equipped with the Plan Apochromat 63 × /1.40 Oil DIC objective and images were analyzed by ZEN microscope-software (ZIESS- Oberkochen-Deutschland).

### Flow Cytometry-Expression Analysis

2 × 10^5^ HEK293T cells were seeded in each well of a 96-well plate in DMEM (1X) (Dulbecco's Modified Eagel Meduim) + GlutaMAX with 10% FCS (FCS) and 1% Penicillin/Streptomycin 24 h prior to transfection. Cells were transfected with pCR3 constructs expressing wildtype-TRAIL and the p.Gln27Lys TRAIL form. Forty eight hours post-transfection cells were harvested and washed two times in ice-cold PBS. Cells were re-suspended in ice-cold PBS containing 1:500 ratios of Fixable viability Dye (eFluor 506 - # 65086614 eBioscience- Darmstadt-Germany) and incubated 30 min at 4°C. Incubated cells were divided in to two groups. The first group was used for the surface staining and washed with ice-cold FACS buffer (PBS with 5% FCS) and incubated with monoclonal PE anti-human CD253 (TRAIL) antibody (#308205-Biolegend- San Diego, CA 92121) 30 min at 4°C and finally re-suspended in 200 μl of FACS buffer and analyzed by BD FACSCanto™ II system. The rest of the cells, used for the internal staining, washed, fixed and permeablized with 1X BD cytofix/Cytoperm solution (#51-2090KZ-Heidelberg Germany) and BD Perm/wash (#51-20901KZ- Heidelberg Germany) buffer, respectively, based on the manufacturer (BD-Bioscience). Fixed cells were incubated with monoclonal PE anti-human CD253 (TRAIL) Antibody (#308205-Biolegend- San Diego, CA 92121) for 30 min at 4°C and later re-suspend in FACS buffer and the TRAIL expression was analyzed by BD FACSCanto™ II system.

### Viability and Apoptosis Analysis-Annexin-V

2 × 10^5^ HEK293T cells were seeded in each well of a 96-well plate in RPMI Medium 1640 (1X) with 10% FCS and 1% Penicillin/Streptomycin 24 h prior to transfection. Cells were transfected with pCR3 constructs expressing wildtype-TRAIL and p.Gln27Lys mutant forms. Forty-eight hours later, transfected cells were irradiated 30–45 min by with 10 γ-rays (40 Gray). The co-culture model was established by adding 2 × 10^5^ of BJAB cells (EBV-negative Burkitt-like lymphoma cell line) to the previously transfected HEK293T cells in the same plate. As a positive control for apoptosis, in one well only BJAB cells were seeded and treated with 180 μM of H2O2. BJAB cells which were not in co-culture with HEK293T cells and did not exposed to any kind of treatment were considered as the negative control. The co-cultured cells were incubated at 37°C and after 24 h. First, the BJAB cells were collected, washed and stained with anti-human IgM-PerCp-Cy5.5 (#314512-Biolegend- San Diego, CA 92121) incubated for 15 min in dark. Cells were washed three times FACS buffer and 7AAD viability dye was added to each sample prior to analysis by BD FACS Canto™ II system. HEK293T cells were harvested and by 0.25% Trypisin-EDTA (1X) (#25200056 Life Technologies-Darmstadt-Germany) and washed with ice-cold FACS buffer (PBS with 5% FCS) and incubated with monoclonal PE anti-human CD253 (TRAIL) antibody (#308205-Biolegend- San Diego, CA 92121) 30 min at 4°C and finally washed and re-suspended in 200 μl of FACS buffer and the surface expression of TRAIL was analyzed by BD FACS Canto™ II system. In another approach, we used jurkat cells (T cell leukemia cell line) as the target of apoptosis to analyze the effect of p.Gln27Lys mutation. The co-culture model was established by adding 2 × 10^5^ of Jurkat cells to the equal number of transfected HEK293T cells. After 24 h, Jurkat cells were collected and the apoptosis analysis was performed by FITC-AnnexinV Apoptosis Detection Kit II (#556570-BD Biosciences- Heidelberg Germany) and Dapi as the viability dye.

### NFκB Activation-Dual Luciferase Reporter Assay

NFkB detection was performed as previously described (44, 45). Briefly, 5 × 10^5^ HEK293T cells were seeded in a 96 well plate in DMEM (1X) (Dulbecco's Modified Eagel Meduim) + GlutaMAX with 10% FCS and 1% Penicillin/Streptomycin 24 h prior to transfection. Cells were transfected with a mix of vectors containing: EGFP (transfection efficiency control) (7 ng), control renilla vector (7 ng), NF-κB firefly luciferase reporter vector (7 ng) and increasing concentrations of the wildtype and p.Gln27Lys TRAIL, ranging from 0.05 to 12.8 ng (70 ng/well total DNA), using *jetPEI* transfection kit (#101-01N, Polyplus, Illkirch France). Twenty four hours later, cells were lysed and expression of firefly and renilla luciferases was detected with the dual luciferase assay detection kit (Promega, MA). The NFκB activation fold was measured via the chemiluminescence reaction by EnVision 2105 Multimode Plate Reader (#2105-0010 -PerkinElmer- Hamburg Germany).

## Discussion

Mutations in *IKZF1* encoding the transcription factor IKAROS have been previously described in 42 CVID patients and shown to contribute to the disease phenotype (16, 34–37). In this study, we identified seven heterozygous missense variants, one frame-shift and one truncating mutation in 13 patients of nine unrelated families in our PID cohort. By transient overexpression we showed that all identified IKAROS variants analyzed in this study, can gained expression levels comparable to the wildtype protein, consistent with a previous report (16). As a transcription factor, the ability of IKAROS to bind to the regulatory elements of its target genes is crucial for its normal function. Direct DNA binding is the major mechanism by which IKAROS is targeted to foci of pericentromeric-heterochromatin (19). In analogy with previously reported mutations (16), the p.Arg143Trp variant and the experimentally generated p.Arg162Gln variant, which both affected the DNA binding domain of IKAROS, as well as the p.Cys150Arg which affects the Zn^2^^+^ binding site in the DNA binding region, failed to form foci with PC-HC localization and were unable to bind IKBS-4 which contains a high-affinity IKAROS binding site derived from binding site selection studies (46). Interestingly, the loss of the DNA binding ability in EMSA and the aberrantly diffuse sub-nuclear localization of mutant IKAROS was reversed, when cells were co-transfected with both equal amounts wildtype and mutant form. These results suggest that the DNA binding ability of wildtype IKAROS was not prohibited by these three mutations and p.Arg143Trp and p.Cys150Arg do not have dominant negative effects. The two C-terminal zinc finger motifs of IKAROS, forming the dimerization domain, are required for its normal binding to the target DNA as a homodimer (19, 47), and the linker regions between zinc finger motifs have important regulatory functions for IKAROS activity, since they contained several phosphorylation sites which can be targeted by casein kinase 2 or Bruton's tyrosine kinase (48, 49). Phosphorylation of the linkers regions is a potentially important mechanism for the regulation of transcriptional activity of IKAROS (48). In our study, none of the phosphorylation sites (Ser214 and Ser215 for Bruton's tyrosin kinase; Ser13, Thr23, Ser63, Ser101, Met249, and Ser389-Thr398 for Casein kinase 2) were directly affected by the identified variants. Although the truncated variant p.Lys286^*^, affecting the linker region between ZF4 and ZF5, was still able to translocate to the nucleus and also bind to the targeted DNA sequence in EMSA, it showed a diffused pattern of localization which remained impaired upon co-transfection with the wildtype counterpart, whereas DNA binding was unaffected. The retained ability of truncated IKAROS to bind DNA might be dependent on the target sequence (IKBS-4, the default site to which IKAROS dimers bind) which was derived from a pericentromeric region (19). We did not test other DNA-binding motifs such as gamma-satellite DNA, which is present at centromeric structures and within heterochromatin as well as in pericentromeric regions of human chromosomes 8, X, and Y (50–52). Since the gamma-satellite DNA forms huge 10- to 200-kb clusters, due to its tandem array of 220 bp GC-rich repeat units (51), it is possible that the truncated IKAROS variant p.Lys286^*^ lost the ability to form pericentromeric DNA-protein complexes with the tandem repeats in the satellite DNA at pericentromeric regions (becoming apparent as punctate patterns in microscopic analyses) although still able to bind the IKBS-4 motif. The mutation p.Met494Val, which is in the short linker between ZF5 and ZF6, showed an impaired oligomerization when co-expressed with the wildtype-IKAROS construct; however, it was still able to bind as a dimer to the target sequence in the EMSA. The pericentromeric-heterochromatin localization of the p.Met494Val IKAROS variant was unaffected. Therefore, IKAROS missense variants affecting the DNA binding domain are most likely loss-of-function variants, but without exerting dominant negative effects. A functional defect of the remaining IKAROS variants could not be evaluated with the tests implemented for this study (Table 2). Thus, based on the data described above, an impaired DNA binding ability and pericentromeric-heterochromatin localization defect of IKAROS variants was only observed in four out of the nine variants (p.Arg143Trp in Family A; p.Met494Val in Family D; p.Cys150Arg and p.Lys286^*^ in Family E and G, respectively), analyzed in this study. Further functional studies on IKAROS responsive genes may shed light on the impact of all these mutations at a transcriptional level.

**Table 2:** Summary of the results.

** 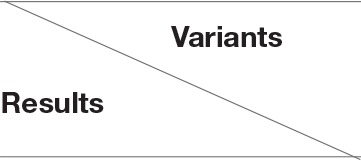 **	**Wildtype**	**R143W**	**C150R**	**R162Q (experimental control)**	K286^*^	**G337S**	**A448R**	**M494V**	**H119R**	**V139A**	**M494fs**
DANN score	–	0.9993	0.9976	–	0.9956	0.5549	–	0.9686	0.2877	0.4585	–
GERP (NR)	–	6.0799	6.0799	–	5.1999	5.1999	–	5.75	3.73	4	–
Proven score	–	−6.932	−10.570	−3.520	–	1.872	−2.441	−0.691	1.437	0.562	–
Proven prediction (cutoff:-2.5)	–	Deleterious	Deleterious	Deleterious	–	Neutral	Neutral	Neutral	Neutral	Neutral	–
Mutation taster	–	Disease causing	Disease causing	–	Disease causing	Disease causing	–	Disease causing	Polymorphism	Polymorphism	Disease causing
FATHMM	–	Tolerated	Damaging	–	–	Tolerated	–	Tolerated	–	–	–
FATHMM-MKL	–	Damaging	Damaging	–	Damaging	Neutral	–	Damaging	Neutral	Neutral	–
MetaSVM	–	Tolerated	Damaging	–	–	Tolerated	–	Tolerated	Tolerated	Tolerated	–
The expression of full-length IKAROS protein in the nuclear extract in western blotting	Expressed	Expressed	Expressed	Expressed	Not expressed^a^	Expressed	Expressed	Expressed	Not done	Not done	Not done
The expression of full-length IKAROS in wildtype/mutant co-transfected cells in western blotting	Expressed	Expressed	Expressed	Expressed	Expressed	Expressed	Expressed	Expressed	Not done	Not done	Not done
DNA/Protein complexes formation in wildtype or mutant IKAROS in EMSA	DNA/Protein bands were observed	DNA/Protein bands were absent	DNA/Protein bands were absent	DNA/Protein bands were absent	DNA/Protein bands were observed	DNA/Protein bands were observed	DNA/Protein bands were observed	DNA/Protein bands were observed	Not done	Not done	Not done
DNA/Protein complexes formation in wildtype/mutant co-transfected cells in EMSA	DNA/Protein bands were observed	DNA/Protein bands were observed	DNA/Protein bands were observed	DNA/Protein bands were observed	DNA/Protein bands were observed	DNA/Protein bands were observed	DNA/Protein bands were observed	DNA/Protein bands were observed	Not done	Not done	Not done
Pericentromeric-Heterochromatin localization(punctate staining pattern) in wildtype or mutant IKAROS	Punctate pattern observed	Diffused staining pattern was observed	Diffused staining pattern was observed	Diffused staining pattern was observed	Diffused staining pattern was observed	Punctate pattern observed	Punctate pattern observed	Punctate pattern observed	Not done	Not done	Not done
Pericentromeric-Heterochromatin localization(punctate staining pattern) in wildtype/mutant co-transfection	Puctate pattern observed	Puctate pattern observed	Puctate pattern observed	Puctate pattern observed	Speckled pattern was formed mainly by the wildtype construct^b^	Puctate pattern observed	Punctate pattern observed	Punctate pattern observed	Not done	Not done	Not done
Molecular defects	–	Loss of function	Loss of function	Loss of function	Loss of function	–	–	–	–	–	–

a A band of 31 kDa was observed in the nuclear extract of cells, transfected with this variant. The full-length IKAROS could not be detected in this variant due to the stop-gained mutation leading the truncation in Lys286^*^.

b The localization defect of the p.Lys286* mutant, is not terminated with co-expression of wildtype IKAROS, consistent with the loss of its dimerization domain. The localization of the co-transfected wildtype IKAROS remained unaffected in these cells.

In our study, the clinical manifestations and laboratory findings varied among patients of different families and even between affected individuals of one pedigree. An increased frequency of transitional B cells was previously reported in five patients carrying *IKZF1* mutations (34, 35). This data could suggest an abnormal B-cell development in patients carrying *IKZF1* mutations and confirmed the critical role of IKAROS in B cell development after leaving the bone marrow. In our study, the increased percentage of transitional B cells was observed only in the affected members of Family A and D (the percentage of IgM^++^ CD38 transitional B cells was not determined for patient E.II.1 and G.II.2). However, the absolute number of transitional B cells in the blood was within the normal range. Thus, it could be possible that the elevated percentage of the transitional B cells could be rather the consequence of a decrease percentage of another lymphocyte population such as CD27 memory B cells. Previous T cell studies in CVID patients with *IKZF1* mutations showed a consistent increase in the number CD8^+^ T cells with reversed CD4:CD8 ratios as the result of increased CD8^+^ T-cell counts (16, 35, 36). In addition, increased number of CD8^+^ T cells was also observed in three CID patients reported by Boutboul and colleagues. However, these patients were reported to have T cell lymphocytosis, affecting both CD4^+^ and CD8^+^ cells (38). Previous animal studies showed that, naive murine CD8^+^ T cells haploinsufficient for *IKZF1* failed to upregulate Ikaros, produced increased amount of autocrine IL-2, and differentiated in an IL-2-dependent manner into IFN-γ-producing CTLs in response to TCR/CD28 stimulation. These data showed that IKAROS regulates CD8^+^ T cell differentiation in mice by restricting autocrine IL-2 production (53). In addition, a T cell specific dominant negative effect in human patients with IKAROS mutations, producing unstable proteins that fail to bind DNA, has been suggested (16). In our study, however, the elevated number of CD8^+^T cells was observed only in patient C.II.1, carrying the Val139Ala variant which was not analyzed in this study. By contrast, patient D.II.2 had a reduced number of CD8^+^T cells. Reduced number of CD8^+^ T cells was previously reported in two CID patients with severe lymphopenia (38). Autoimmune manifestations including Hashimoto thyroiditis, autoimmune cytopenia, and seronegative arthritis were among the clinical manifestations of patients A.I.2, D.II.1, and E.II.1. Somatic loss-of-function and dominant negative mutations in *IKZF1* have been shown to be associated with human B-cell leukemia (54–57). More recently, the missense mutation p.Asn159Ser was shown to be associated with primary immunodeficiency that progressed to T-cell acute lymphoblastic leukemia (37, 38). Regarding malignancies in our cohort, patient G.II.2, carrying the Lys286^*^ mutation, developed pediatric B-ALL. CNS lymphoma was also observed in the affected member of Family D (D.II.2). However, mutations in *IKZF1* have not been reported to be associated with human CNS lymphoma.

The first truncating *IKZF1* mutation associated with IKAROS haploinsufficiency was reported by Bogaert and colleagues in two siblings with different kinds of arthritis and recurrent bacterial sinopulmonary infections. Interestingly, the asymptomatic mother of these two children also carried the same mutation (34). Similarly, in our cohort, four asymptomatic individuals carried *IKZF1* mutations which did not have dominant-negative effects. These results which is suggesting an incomplete penetrance is consistence with the previously published data (16, 34). In general in monogenic causes of PID, haploinsufficiency has been shown to favors incomplete penetrance more than dominant-negative mutations (58). Moreover, CVID is an immunodeficiency with a late age of onset in most cases and clinical manifestations of the disease can appear as late as in the sixth decade of life, therefore it may be that currently asymptomatic carriers may yet develop features of the disease.

In this study we also identified a heterozygous missense variant in *TNFSF10* (TRAIL) in all the affected individuals of Family A. Mutations in *TNFSF10* have not been reported to be associated with a CVID-like phenotype, however, due to the role of TRAIL in regulating B cells responses (59, 60), mutation in this gene were considered as a candidate to modify the CVID phenotype. Human *TNFSF10* is located on chromosome 3q26 and encodes TNF-related apoptosis-inducing ligand (TRAIL). TRAIL is a type II transmembrane protein and contains of 281 amino acids. The N-terminal cytoplasmic domain of TNF superfamily is not conserved between members, but the C-terminal extracellular domain is conserved and can be cleaved proteolytically from the cell surface (61, 62). TRAIL is expressed in various tissues, especially in the lung, prostate and spleen and different cells of the immune system. Both soluble and membrane-bound TRAIL is expressed by activated NK and CD8^+^ T cells as well as monocytes and CpG-stimulated B cells (63, 64). TRAIL can bind to two different types of receptors: death receptors which induce apoptosis in the target cell and decoy receptors which inhibit this pathway. Four human specific receptors for TRAIL have been recognized: the death receptors TRAIL-R1 (DR4) and TRAIL-R2 (DR5) and the decoy receptors TRAIL-R3 (DcR1) and TRAIL-R4 (DcR2). TRAIL can also bind to the soluble osteoprotegerin (OPG) (61). Upon binding to its death receptors, DR4 and DR5, TRAIL can trigger the apoptosis pathway by recruiting Fas-associated protein with death domain (FADD) and lead to the direct activation of the caspase cascade resulting in apoptotic cell death (65). Paradoxically, TRAIL binding to its death receptors, activates NF-kB and promotes the transcription of genes that induce cell survival and resistance to apoptosis (66).

Our data showed that the NFκB activation pathway downstream of TRAIL and TRAIL-induced apoptosis were affected in HEK293T cells expressing the p.Gln27Lys variant, identified in Family A. Moreover, the results of the expression analysis by flow cytometry revealed that the surface localization of TRAIL was inhibited by the p.Gln27Lys, affecting the transmembrane region of the protein. Thus, the reduced NFκB activation as well as impaired apoptosis in cell expressing p.Gln27Lys could be the results of reduced surface expression of the ligand and the consequent accumulation of the protein in ER.

Although previously described familial cases of CVID suggested rare monogenic causes, a majority of CVID cases are thought to be polygenic in origin (67). For instance, in 2011, 363 CVID patients were evaluated by a genome-wide association study (GWAS) for single nucleotide polymorphisms (SNPs) in 610,000 genes. The results revealed a strong association with a disintegrin and metalloproteinase (ADAM) gene regions and major histocompatibility complex (MHC) regions (67, 68). This data suggests that the CVID-like phenotype can be the result of mutations in more than one gene and the heterogeneity of the disease and the variability in the occurrence of autoimmunity and other manifestations may be explained by the polygenic origin of CVID. Thus, the *IKZF1* mutation p.Arg143Trp, identified in Family A was shown to impair the function of IKAROS, though it can also be possible that the CVID-like phenotype in this family can be the results of the contribution of *IKZF1* mutation and the missense variant identified in *TNFSF10*.

Taken together, in this study we identified nine germline heterozygous *IKZF1* variants in our PID cohort. Three of the identified mutations, p.Arg143Trp, p.Cys150Arg, and p.Lys286^*^ led to the disruption of IKAROS function and can explain the CVID phenotype with hypogammaglobinemia and B-cell defects *via* impairing the DNA binding ability of IKAROS and consequently the transcriptional regulation of B cell development and function. Further investigations, particularly B cell studies in patients' samples, are needed to find the exact correlation of these mutations with B cell defects and CVID-like phenotype. In future work, it is also important to understand the effects of the identified *TRAIL* mutation in Family A and investigate its possible role in B cell defects and antibody deficiency and its contribution to CVID-like phenotype.

## Data Availability

Data have been uploaded to ClinVar, accession number: SUB5336620.

## Ethics Statement

This study was conducted under the approved ethics protocol no 295/13 for human subjects. Samples were collected with the written consent of all study participants and/or their parental guardians after formal ethical approval by the local ethics committees at the University Hospital of Freiburg and collaborating institutes.

## Author Contributions

ZE performed all the experiments, analyzed the data, prepared all the figures and wrote the first draft of the manuscript. MF designed the cloning primers, helped with performing confocal microscopy, and revised the content of the paper. AB analyzed the data of WES and targeted gene panel sequencing and wrote the genetic analysis section of the manuscript. MP established and designed the co-culture system for the TRAIL project. RH provided more detailed explanations about the clinical manifestation and lab results of Family G. CS established the NFKB activation reporter assay and helped for interpreting the data. DS helped with the expression analysis of TRAIL and data statistical analysis. KW revised the paper critically for the content and provided and cared for study patients (patients of Family A and Family F). BG performed conception and designing of the study, provided funding for the whole project and provided and cared for study patients and revised the paper critically for the intellectual content. All the authors have received and read the last version of the manuscript before submission.

### Conflict of Interest Statement

The authors declare that the research was conducted in the absence of any commercial or financial relationships that could be construed as a potential conflict of interest.
