# Corrigendum: Assessing the Functional Relevance of Variants in the *IKAROS Family Zinc Finger Protein 1* (*IKZF1*) in a Cohort of Patients With Primary Immunodeficiency

**DOI:** 10.3389/fimmu.2019.01490

**Published:** 2019-06-28

**Authors:** Zoya Eskandarian, Manfred Fliegauf, Alla Bulashevska, Michele Proietti, Rosie Hague, Cristian Roberto Smulski, Desirée Schubert, Klaus Warnatz, Bodo Grimbacher

**Affiliations:** ^1^Institute for Immunodeficiency, Center for Chronic Immunodeficiency, Medical Center, Faculty of Medicine, Albert-Ludwigs-University of Freiburg, Freiburg, Germany; ^2^Faculty of Biology, Albert-Ludwigs-University of Freiburg, Freiburg, Germany; ^3^Centre for Integrative Biological Signalling Studies, Albert-Ludwigs University of Freiburg, Freiburg, Germany; ^4^Royal Hospital for Children, Glasgow, United Kingdom; ^5^Department of Medical Physics, Centro Atómico Bariloche, CONICET, San Carlos de Bariloche, Argentina; ^6^Clinic for Rheumatology and Clinical Immunology, Faculty of Medicine, CCI, Medical Center, Albert-Ludwigs-University of Freiburg, Freiburg, Germany; ^7^Satellite Center Freiburg, RESIST–Cluster of Excellence 2155, Hanover Medical School, Freiburg, Germany; ^8^Satellite Center Freiburg, German Center for Infection Research, Freiburg, Germany; ^9^Institute of Immunity and Transplantation, Royal Free Hospital, University College London, London, United Kingdom

**Keywords:** CVID, monogenic defects, IKAROS, TRAIL, DNA binding, nuclear localization

In the published article, there was an error regarding the affiliations for “Bodo Grimbacher.” As well as having affiliations 1, 3, 7 and 8, they should also have the “Institute of Immunity and Transplantation, Royal Free Hospital, University College London, London, United Kingdom.”

The authors apologize for this error and state that this does not change the scientific conclusions of the article in any way. The original article has been updated.

