# Supplementary figures and images for: Assessing the Functional Relevance of Variants in the IKAROS Family Zinc Finger Protein 1 (IKZF1) in a Cohort of Patients With Primary Immunodeficiency
Source: Front Immunol. 2019 Apr 16;10:568. doi: 10.3389/fimmu.2019.00568 (PMC6477086; doi:10.3389/fimmu.2019.00568)

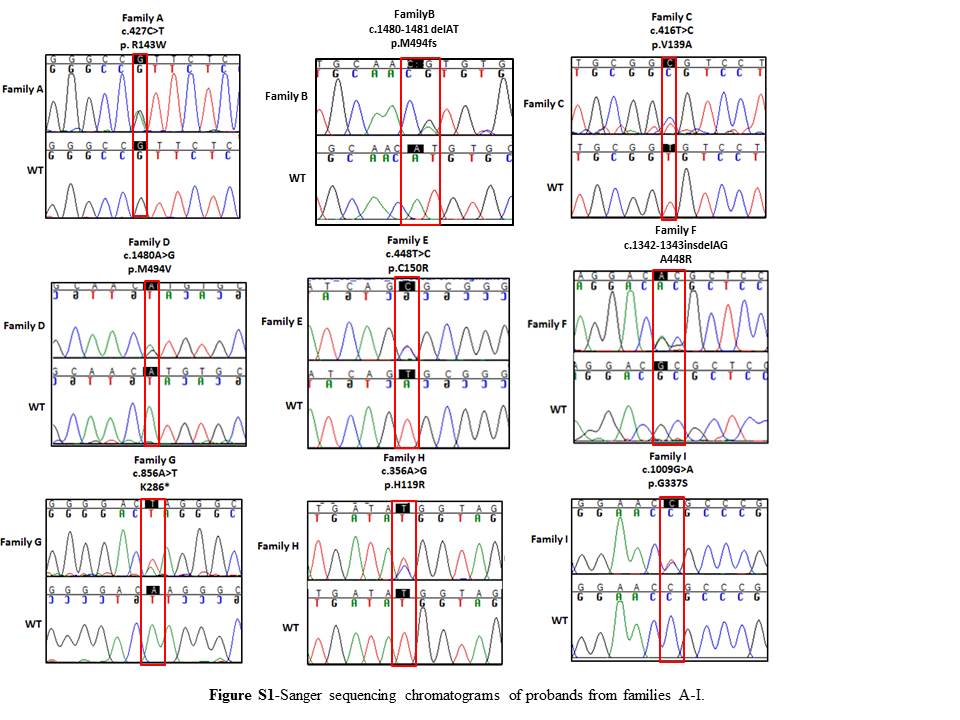

Supplement: Supplementary file 1 [file Image_1.JPEG]

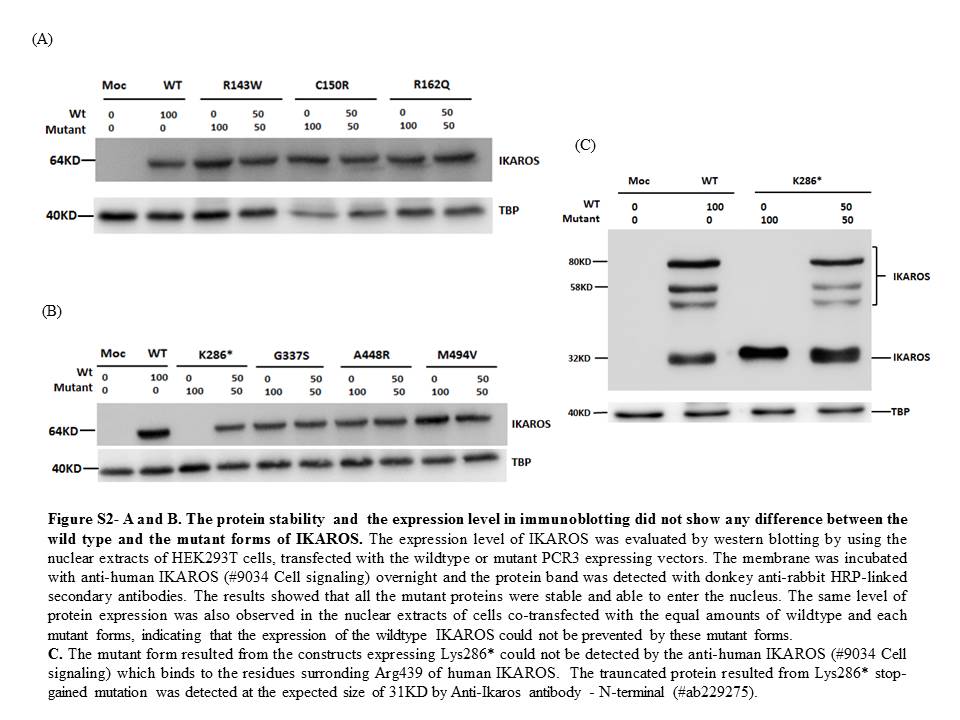

Supplement: Supplementary file 2 [file Image_2.JPEG]
