# Supplementary material for: Assessing the Functional Relevance of Variants in the IKAROS Family Zinc Finger Protein 1 (IKZF1) in a Cohort of Patients With Primary Immunodeficiency
Source: Front Immunol. 2019 Apr 16;10:568. doi: 10.3389/fimmu.2019.00568 (PMC6477086; doi:10.3389/fimmu.2019.00568)
Supplement: Supplementary file 3 [file Image_3.pdf]

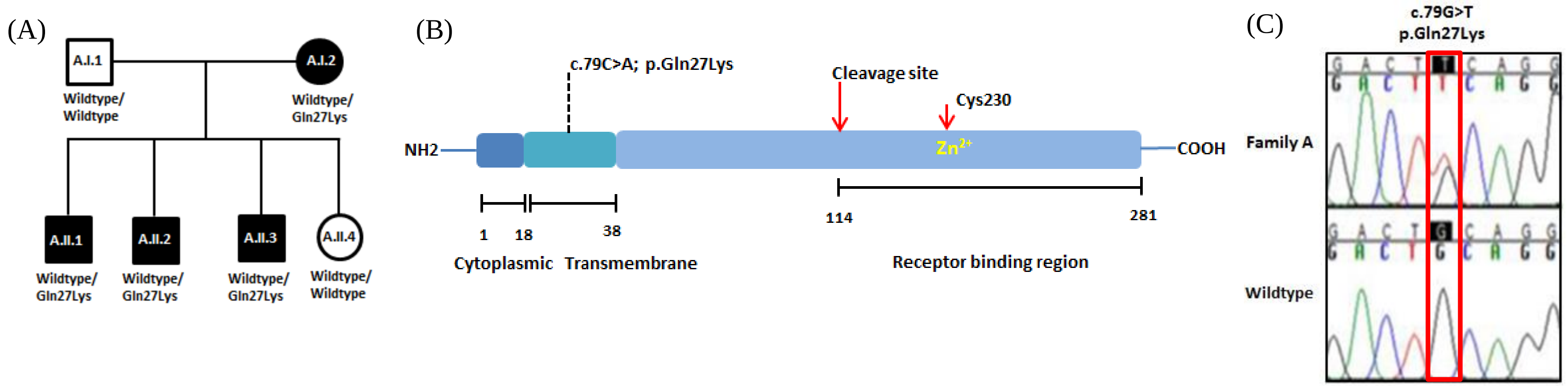

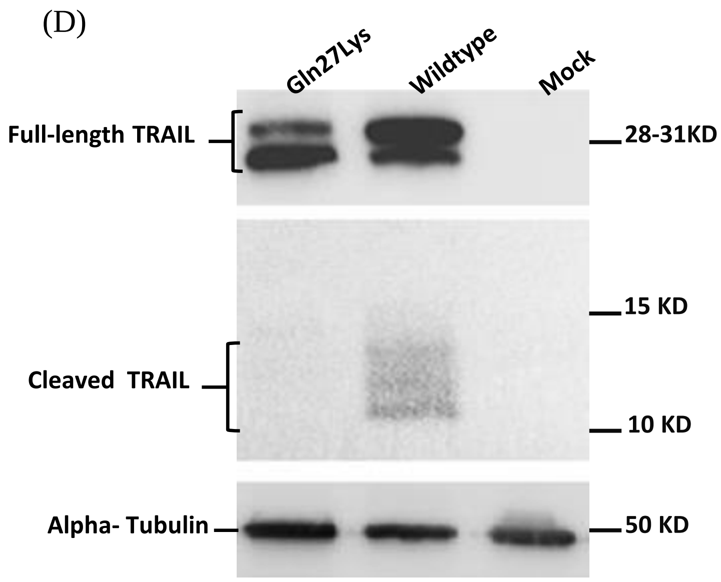

Figure S3-A) Pedigree of family A. Squares and circles indicate male and female subjects, respectively. Filled symbols, affected individual; open symbol; unaffected individual. **B) Sanger sequencing.** Chromatograms of the identified mutation in *TNFSF10* in the affected individuals of Family A. **C) Schematic representation of the structure of the full-length TRAIL protein.** Dotted lines indicated the mutation identified in Family A. Red arrows represented the cleavage site and the Cysteine residue which is important for chelating the Zn^{2+} atom. Numbers indicate amino acids residues. **D) Glycosylation pattern was affected and the cleavage of TRAIL was impaired by the p.Gln27Lys variant.** Western blotting was performed by the cytoplasmic extract of HEK293T cells transfected with wildtype or p.Gln27Lys-pCR3-TRAIL expression vectors. Upper panel) two sharp bands were observed at the expected size of 28-31 kDa in the whole lysate of cells expressing wildtype and the mutant forms. However, the larger band corresponding to the glycosyzilated form of TRAIL was weaker in cells expressing p.Gln27Lys TRAIL. Middle panel) Bands observed between 10-15 kDa represented the residues resulted from the cleavage form of the membrane-bound TRAIL. These bands were undetectable in cells transiently transfected with the construct expressing p.Gln27Lys TRAIL. Lower panel) Equal protein loading was assured by α-Tubulin level.

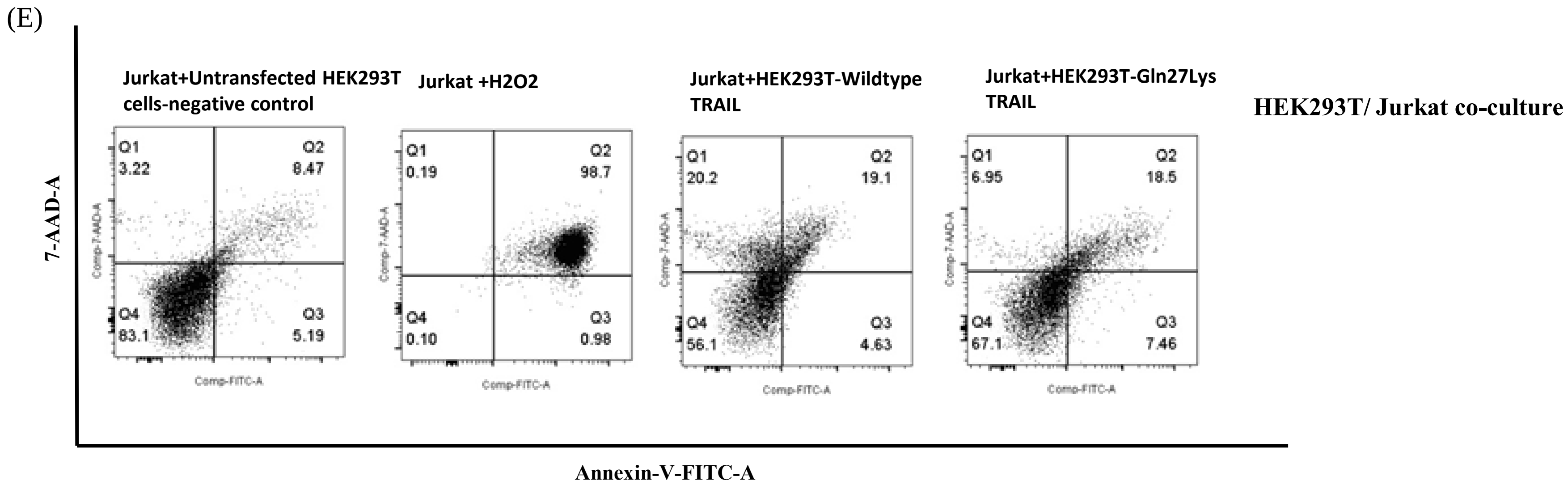

Figure S3- E) Delayed TRAIL-induced apoptosis in Jurkat cells, co-cultured with HEK293T cells expressing p.Gln27Lys form. Jurkat cells were co-cultured with HEK293T cells, transiently transfected with wildtype or mutant TRAIL. Apoptosis was measured by annexin-V-FITC analysis. H_2O_2-induced apoptosis was used as a positive control.

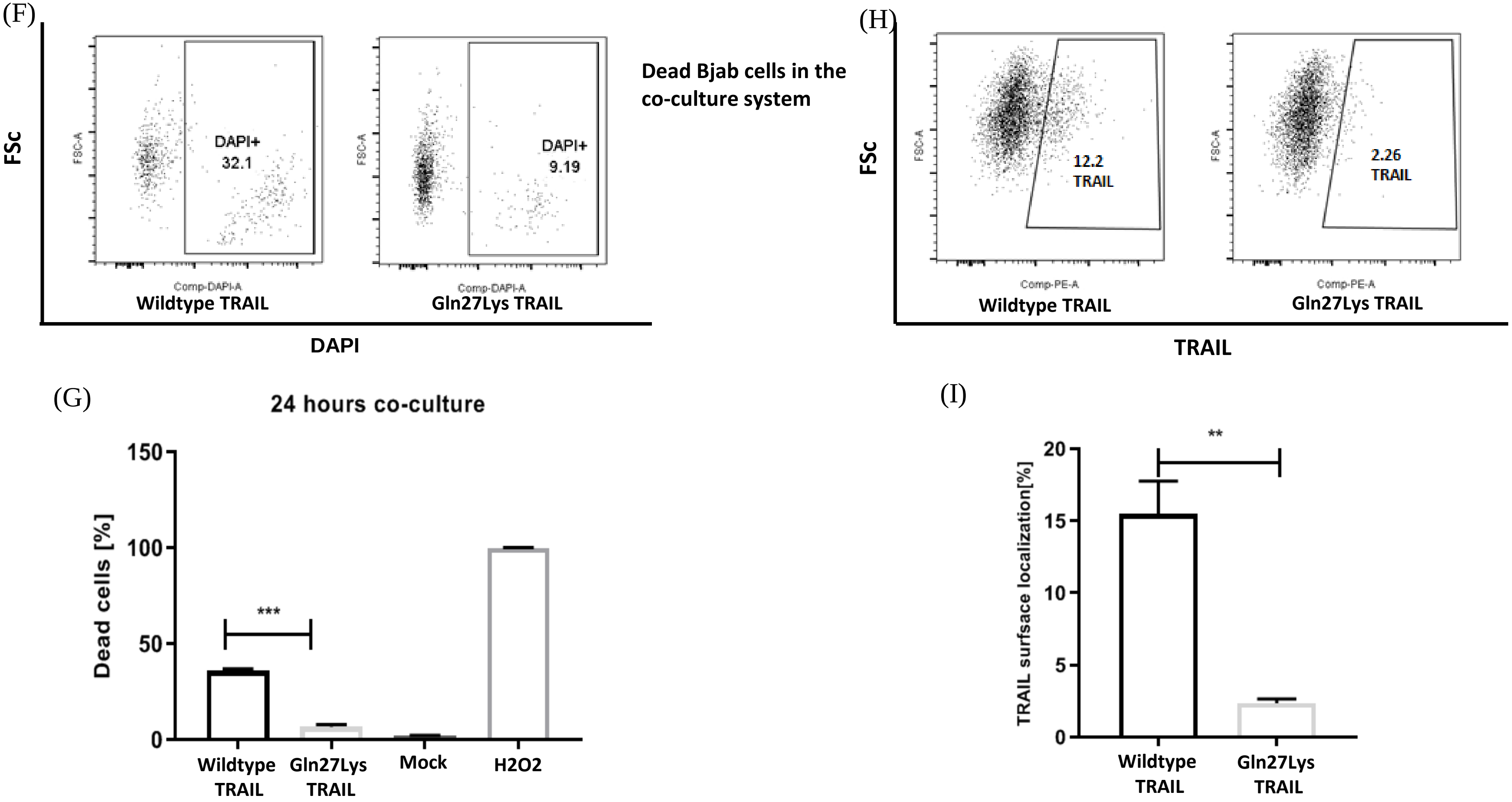

Figure S3-F and G) The percentage of viable Bjab cells was significantly higher in the co-culture system of Gln27Lys TRAIL. The cell death induction by TRAIL was evaluated by DAPI viability dye staining in a co-culture system including IgM$^+$Bjab cells and HEK293T cells transiently expressing the wildtype TRAIL or the mutant form. The percentage of dead cells among IgM$^+$Bjab cells, co-cultured with p.Gln27Lys was significantly lower than cells cultured with cells expressing the wildtype TRAIL, indicating the impairment of cell-death induction by cells overexpressing the p.Gln27Lys TRAIL. **H and I) Reduced surface expression of mutant TRAIL in co-cultured HEK293T.** HEK293T cells derived from the co-culture were analyzed for surface expression of wildtype and mutant p.Gln27Lys TRAIL by flow cytometry.

(J)

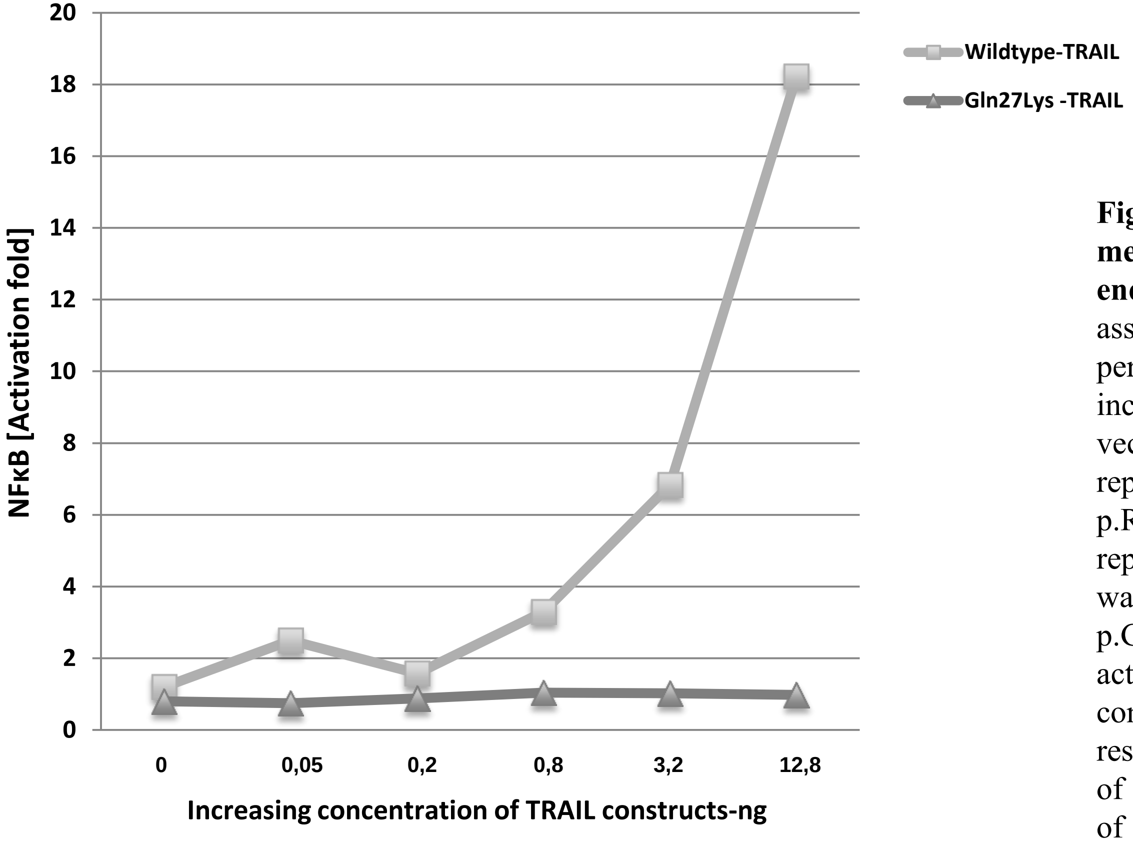

(K)

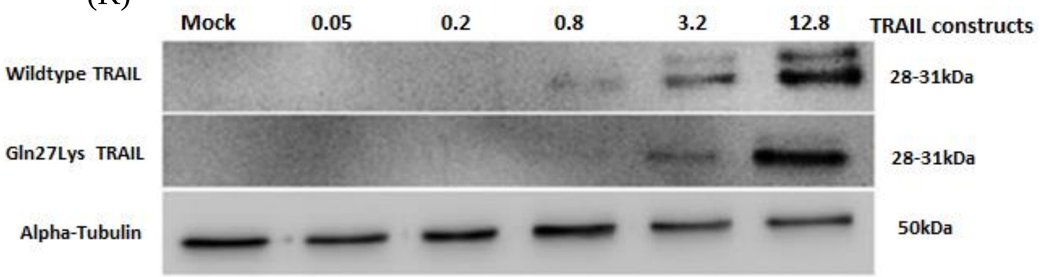

Figure S3-J) p.Gln27Lys TRAIL was unable to mediate activation of the reporter via the endogeneous NF-κB. A NF-κB Dual luciferase reporter assay (#E1910 Promega-Mannheim Germany) was performed by using HEK293T cells, co-transfected with increasing amounts of wildtype or mutant pCR3-TRAIL vector constructs, ranging from 1.6 ng to 12.8 ng and a reporter vector (p.NFκB-luciferase vector construct) and p.Renilla vector construct as an internal control reporter. The results showed that, the NFκB activation was severely impaired in cells overexpressing the p.Gln27Lys mutant and the pathway could not be activated even in cells transfected with the highest concentration (12.8ng) of the TRAIL construct. **K)** The results of western blotting with the remaining cell lysate of the reporter assay revealed that the expression level of p.Gln27Lys was comparable with the wildtype one, but the band of 31kDa, indicating the glycozylated form of TRAIL, was absent in the lysate of p.Gln27Lys TRAIL expressing cells.

(L)

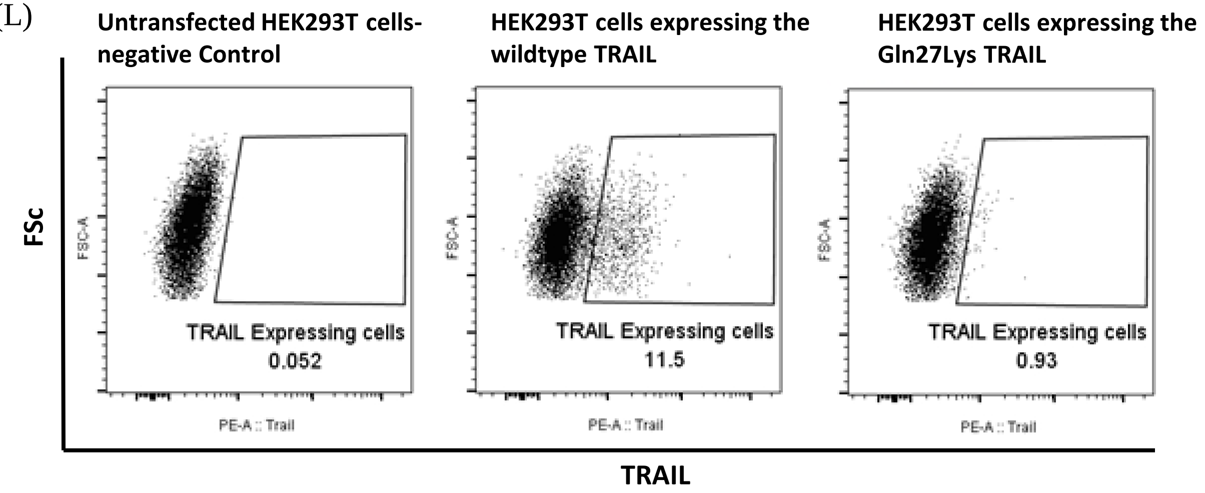

Figure S3-L) The missense mutation affecting the transmembrane region of the protein inhibited the surface localization of TRAIL. HEK293T cells were transfected with wildtype or mutant construct of TRAIL. Cells were stained with monoclonal PE anti-human CD253 (TRAIL) antibody and the surface expression of the protein was measured with flow cytometry, forty eight hours post-transfection. **M)** The significant reduction of surface expression was observed in cells overexpressing the p.Gln27Lys, compare to the wildtype. **N)** Similarly, the ratio of surface to the total TRAIL expression was remarkably lower in HEK293T cells expressing this mutant form.

(M)

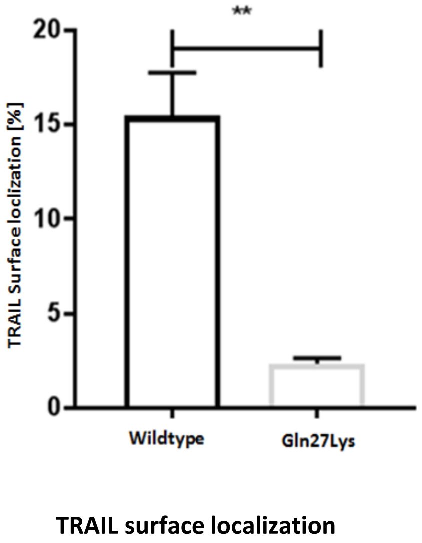

(N)

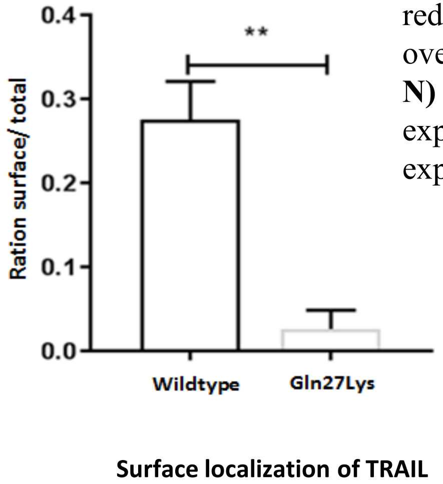

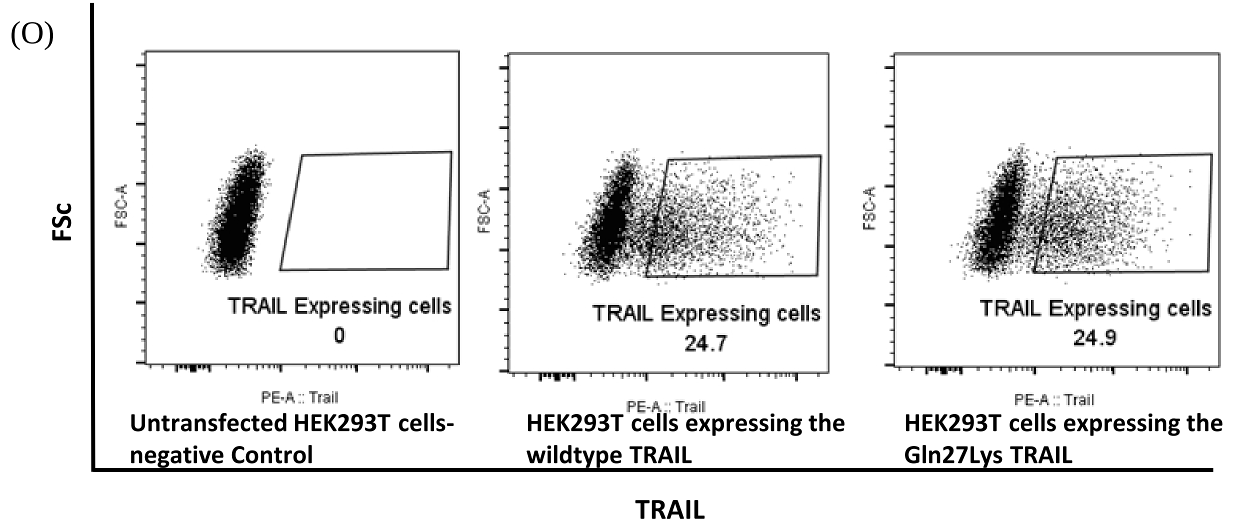

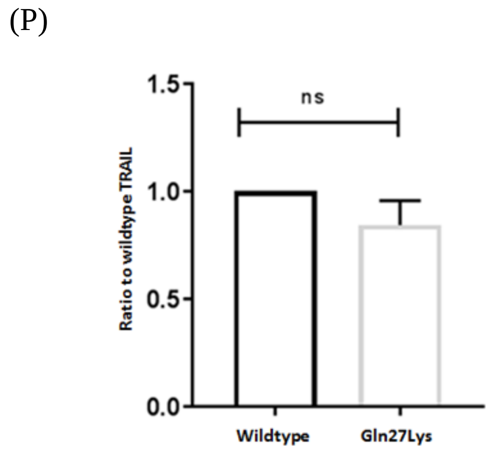

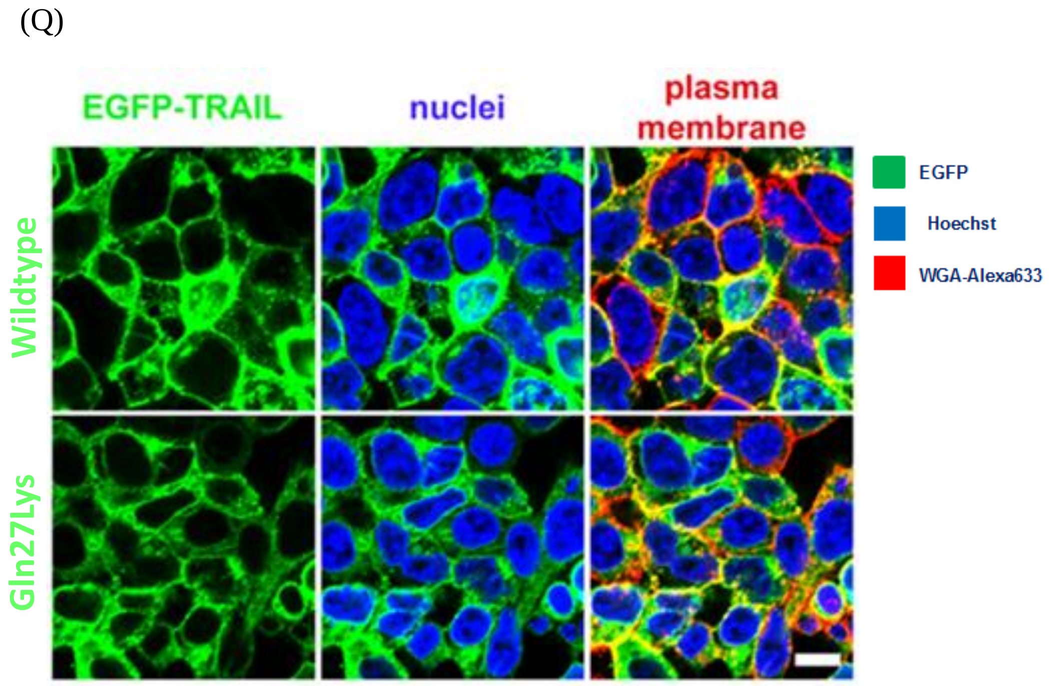

Figure S3-O and P) The p.Gln27Lys mutation impaired the surface localization of TRAIL and caused protein accumulation in ER. The internal staining with PE anti-human CD253 (TRAIL) showed no significant difference between cells expressing p.Gln27Lys mutant form and those transfected with the wildtype construct, indicating that the p.Gln27Lys TRAIL was mainly accumulated in the ER and failed to reach the cell surface. **Q) Reduced surface localization of p.Gln27Lys-TRAIL.** HEK293T cells were transfected with pEGFP-C1 vector constructs expressing EGFP-fused wildtype or p.Gln27Lys mutant TRAIL. Upper panel) EGFP- TRAIL-wildtype was predominantly localized to the plasma membrane in transfected cells (please note the EGFP-negative space between nuclei and plasma membrane). Lower panel) pEGFP-p.Gln27Lys-TRAIL shows reduced plasma membrane localization but an increase and diffuse cytoplasmic distribution.
