# Supplementary material for: Assessing the Functional Relevance of Variants in the IKAROS Family Zinc Finger Protein 1 (IKZF1) in a Cohort of Patients With Primary Immunodeficiency
Source: Front Immunol. 2019 Apr 16;10:568. doi: 10.3389/fimmu.2019.00568 (PMC6477086; doi:10.3389/fimmu.2019.00568)
Supplement: Supplementary file 4 [file Table_1.pdf]

Table S1. List of oligonucleotides primers used for cloning.

	Name	5' to 3' sequence
1	IKAROS cloning primer- Forward	GATCAAGCTTCAGCCACCATGGATGCTGATGAGGGTCAAGAC
2	IKAROS cloning primer- Reverse	GATCGGATCCTTAGCTCATGTGGAAGCGGTGCTCCCCTCGCG
3	IKAROS-FLAG-Reverse	GATCGGATCCCTACTACTTGTCATCGTCGTCCTTGTAATCGCTCATGT GGAAGCGGTGCTCCCCTCGCG
4	IKAROS-myc-Revers	GATCGGATCCCTACAGATCTTCTTCAGAAATAAGTTTTTGTTCGCTCA TGTGGAAGCGGTGCTCCCCTCGCG
5	R143W mutation-Forward	GAAGCCACACTGGAGAATGGCCCTTCCAGTGCAATC
6	R143W mutation-Reverse	CTTCGGTGTGACCTCTTACCGGGAAGGTCACGTTAG
7	M494V mutation-Forward	ATCCTTTTGAGTGCAACGTGTGCGGCTACCACAGCCAG
8	M494V mutation-Reverse	CTGGCTGTGGTAGCCGCACACGTTGCACTCAAAAGGAT
9	C150R mutation-Forward	TTCCAGTGCAATCAGCGCGGGGCCTCATTCACC
10	C150R mutation-Reverse	GGTGAATGAGGCCCCGCGCTGATTGCACTGGAA
11	A448R mutation-Forward	ACTCGCAGGACAGGCTCCGCGTGGTCAGC
12	A448R mutation-Reverse	GCTGACCACGCGGAGCCTGTCCTGCGAGT
13	K286* G mutation-Forward	GATCAAGCTTCAGCCACCATGGATGCTGATGAGGGTCAAGAC
14	K286* G mutation-Reverse	GATCGGATCCCTAGTCCCCAAGAAATTTCTGAGGCATAGAGC
15	G337S mutation-Forward	GTGCAGACGCCCCCGGGCAGTTCCGAGGTGGTCCCGG
16	G337S mutation-Reverse	CCGGGACCACCTCGGAACTGCCCGGGGGCGTCTGCAC
17	R162Q- Cloning primer-Forward	AGGGCAACCTGCTCCAGCACATCAAGCTGCATTC
18	R162Q- Cloning primer-Reverse	TCCCGTTGGACGAGGTCGTGTAGTTCGACGTAAG
19	TRAIL cloning primer- Forward	GATCAAGCTTCCATGGCTATGATGGAGGTCC
20	TRAIL cloning primer- Reverse	GATCTCTAGATTAGCCAACTAAAAAGGC
21	Q27K TRAIL -Forward	GATCTTCACAGTGCTCCTGAAGTCTCTCTGTGTGG
22	Q27K TRAIL –Reverse	CCACACAGAGAGACTTCAGGAGCACTGTGAAGATC
